# LncRNA TINCR impairs the efficacy of immunotherapy against breast cancer by recruiting DNMT1 and downregulating MiR-199a-5p via the STAT1–TINCR-USP20-PD-L1 axis

**DOI:** 10.1038/s41419-023-05609-2

**Published:** 2023-02-01

**Authors:** Qin Wang, Guozheng Li, Xin Ma, Lei Liu, Jiena Liu, Yanling Yin, Hui Li, Yihai Chen, Xin Zhang, Lei Zhang, Liyang Sun, Jing Ai, Shouping Xu

**Affiliations:** 1 Department of Pharmacology (The State-Province Key Laboratories of Biomedicine-Pharmaceutics of China), College of Pharmacy of Harbin Medical University, 157 Baojian Road, 150086 Harbin, China; 2 Sino-Russian Medical Research Center, Harbin Medical University Cancer Hospital, 150 Haping Road, 150081 Harbin, China; 3 Heilongjiang Academy of Medical Sciences, 157 Baojian Road, 150086 Harbin, China; 4 Department of Breast Surgery, Harbin Medical University Cancer Hospital, 150 Haping Road, 150040 Harbin, China

**Keywords:** Breast cancer, Tumour immunology

## Abstract

Although programmed death-ligand 1 (PD-L1) inhibitors have achieved some therapeutic success in breast cancer, their efficacy is limited by low therapeutic response rates, which is closely related to the immune escape of breast cancer cells. Tissue differentiation inducing non-protein coding RNA (TINCR), a long non-coding RNA, as an oncogenic gene associated with the progression of various malignant tumors, including breast cancer; however, the role of TINCR in tumor immunity, especially in breast cancer, remains unclear. We confirmed that TINCR upregulated PD-L1 expression in vivo and in vitro, and promoted the progression of breast cancer. Next, we revealed that TINCR knockdown can significantly improve the therapeutic effect of PD-L1 inhibitors in breast cancer in vivo. Mechanistically, TINCR recruits DNMT1 to promote the methylation of miR-199a-5p loci and inhibit its transcription. Furthermore, in the cytoplasm, TINCR potentially acts as a molecular sponge of miR-199a-5p and upregulates the stability of USP20 mRNA through a competing endogenous RNA (ceRNA) regulatory mechanism, thus promoting PD-L1 expression by decreasing its ubiquitination level. IFN-γ stimulation activates STAT1 by phosphorylation, which migrates into the nucleus to promote TINCR transcription. This is the first study to describe the regulatory role of TINCR in breast cancer tumor immunity, broadening the current paradigm of the functional diversity of TINCR in tumor biology. In addition, our study provides new research directions and potential therapeutic targets for PD-L1 inhibitors in breast cancer.

## Introduction

In women, breast cancer is the most common malignant tumor and the leading cause of cancer-related mortality worldwide [1]. Breast cancer has been divided into diverse molecular subtypes based on the expression of estrogen receptor (ER), progesterone receptor (PR), and human epidermal growth factor 2 (HER-2) [2]. Although treatment strategies have been personalized to cancer type, the prognosis of breast cancer patients remains unsatisfactory.

Modern immunotherapies, such as PD-L1 inhibitors, have shown promising results. PD-L1 and its receptor, programmed cell death 1 (PD-1), can downregulate T-cell activity and maintain immune tolerance to self-antigens [3, 4]. Similar to the pattern of recognition, cancer cells can escape immune surveillance by upregulating PD-L1 [5]. Increased expression of PD-L1 on the surface of triple-negative breast cancer cells inhibits T-cell proliferation and promotes immune cell apoptosis [6]. Atezolizumab is the first approved PD-L1 monoclonal antibody [7, 8]. Owing to their relatively low response rates, PD-L1 inhibitors are more effective when combined with chemotherapy or other drugs [9–12]. Finding new ways to improve the efficacy of PD-L1 inhibitors has continued to attract attention.

Long non-coding RNAs (lncRNAs), comprising more than 200 nucleotides [13], are involved in multiple physiological and pathological processes [14, 15], especially in cancer development [16]. LncRNAs regulate tumor progression by participating in gene expression, drug resistance, and metastasis [17–20]. Some lncRNAs are positively correlated with lymph node metastasis and can be used as diagnostic biomarkers [21]. Emerging evidence suggests that some lncRNAs play important roles in cancer immunology, including in antigen presentation, immune evasion, and immune cell infiltration [22, 23]. Although many immune-related lncRNAs have been discovered, their functions and specific mechanisms remain elusive.

In this study, we found that TINCR could promote the proliferation and metastasis of breast cancer cells and was positively correlated with PD-L1 expression. Regarding the molecular mechanisms, TINCR potentially promotes miR-199a-5p methylation and acts as a competing endogenous RNA (ceRNA) to upregulate PD-L1 expression by sponging miR-199a-5p. Moreover, IFN-γ promoted the transcriptional expression of TINCR through the upregulation of STAT1. The combination of TINCR knockdown and PD-L1 inhibition showed a synergistic inhibitory effect on breast cancer progression. This study provides novel insights into the roles of PD-L1 inhibitors in the comprehensive management of human breast cancer through immunotherapy.

## Materials and methods

### Breast-tissue specimens and clinical assessments

Patients at the Harbin Medical University Cancer Center (HMUCC) with a histological diagnosis of breast cancer who had not received chemotherapy or radiotherapy before surgical resection were eligible for recruitment to this study. RNA was extracted from breast cancer and normal control tissues stored at −80 °C immediately after resection. Paraffin-embedded tissue sections were produced from tissue samples stored in 4% formaldehyde at 4 °C immediately after resection. This study was approved by and conformed to the clinical research guidelines of the Research Ethics Committee of Harbin Medical University Cancer Hospital. Written informed consent was obtained from all patients.

### Cell culturing, plasmid construction, and transfection

Breast cancer cell lines (UACC812, MDA-MB-231, Hs578T, T47D, BT549, MCF-7 and 4T1) and HEK293T cells were obtained from the Chinese Academy of Sciences Cell Bank and Cellbio. All cell lines were cultured under standard conditions, as specified by the suppliers, in a culture medium supplemented with 100 U/mL penicillin, 100 mg/mL streptomycin, and 10% fetal bovine serum at 37 °C with 5% CO_2_. Mycoplasma testing was performed in-house.

Cells were seeded in six-well culture plates and, at a density of approximately 70%, transfected using JetPrime (#114–15, Polyplus, Germany), according to the manufacturer’s instructions. For lentiviral transduction, we used 4–6 μg/mL polybrene (#107689, Sigma-Aldrich, USA), and 1 μg/mL puromycin (#540411, Calbiochem, USA) was used to select transduced cells. To verify efficiency, cells were harvested for quantitative reverse-transcription polymerase chain reaction (qRT-PCR) analysis. siRNA and plasmids sequences were listed in Tables S1–S2.

### qRT-PCR

Total RNA was extracted from cells and tissue samples using Trizol reagent (#269201, Invitrogen, USA), and 0.4 μg of RNA was reverse-transcribed into cDNA using a cDNA Reverse Transcription Kit (Applied Biosystems, USA). mRNA expression was quantified by real-time PCR using an SYBR Green PCR Master Mix Kit (Applied Biosystems) with gene-specific primers. GAPDH or U6 were used as internal controls, and qRT-PCR was performed on a 7500 FAST Real-Time PCR System (Applied Biosystems). The results were normalized to GAPDH and U6 expression levels using the 2^–ΔΔCt^ method. The primer sequences were listed in Table S3.

### Immunoprecipitation and western blotting

After two washes with ice-cold PBS, 1 × 10^7^ cells were re-suspended in lysis buffer (10 mM Tris-HCl [pH 7.4], 150 mM NaCl, 5 mM NaF, 10 mM DTT, 5% glycerol, and 5000 U/mL proteinase inhibitors) on ice for 1 h and centrifuged at 13,000 × *g* for 7 min. The supernatants were pre-cleared with 40 μL protein A/G-coupled agarose (#sc-2003, Santa Cruz, USA) overnight at 4 °C and then incubated with 5 μg of the indicated antibodies, isotype control IgG-conjugated beads, or 20 μL anti-FLAG affinity gel (#B23101, Bimake, USA) overnight at 4 °C. After three washes with lysis buffer, immunoprecipitates were boiled in 1× loading buffer for western blot analysis.

Cells were lysed with 10 mM Tris-HCl (pH 7.4), 150 mM NaCl, 5 mM NaF, 10 mM DTT, 5% glycerol, 5000 U/mL proteinase inhibitors, and 2% SDS. Protein concentrations were measured using a protein assay kit (#5000001, Bio-Rad, Richmond, CA); equal amounts of protein were separated using SDS-PAGE and transferred onto nitrocellulose membranes blocked with 5% skim milk in TBST for 1 h at room temperature. The primary antibodies against PD-L1 (#104763), USP20 (#132309), Ubiquitin (#128826), STAT1 (#01292), and p-STAT1 (# 50118) were obtained from Gene Tex. After three washes with TBST, the membranes were incubated with HRP-labeled secondary antibodies for 1 h. Then protein bands were visualized using an enhanced chemiluminescence detection system (Western Lightning, Perkin Elmer, Norwalk, CT).

### Immunohistochemistry

Tumor tissues were fixed, embedded, and sectioned (3 μm). In accordance with standard procedures [24], paraffin-embedded tissue sections were subjected to successive deparaffinization, antigen retrieval, background blocking, and target detection with the indicated antibodies. Detection was performed using liquid DAB+ and counterstained with Carazzi’s hematoxylin. The stained sections were independently analyzed by two pathologists.

### Animal experiments

The animal experiments were approved by the Medical Experimental Animal Care Commission of Harbin Medical University. Six-to-eight-week-old female Balb/c mice were obtained from the Beijing Vital River Laboratory Animal Technology Company. Approximately 5 × 10^4^ cells transfected with 4T1-Scramble or 4T1-shTINCR were suspended in 200 μL of serum-free medium. Thereafter, 5 × 10^4^ 4T1 cells were injected into the right mammary fat pad and mice were treated with IFN-γ (300 or 1 000 UI, #S1025, Selleck) orally for 7 d or PD-L1 inhibitor (#751220D1B, Bioxcell, USA) intra-peritoneally administered four times every 4 d at a dose of 75 µg. Tumor growth was measured once every 3 d using calipers, and tumor volume was calculated as 1/2(length × width^2^). After mice were euthanized, injection-induced tumors were excised and weighed.

### Wound-healing assays

Cells were seeded in six-well culture plates on RPMI-1640 or DMEM medium containing 5% FBS and cultivated to a sub-confluent state. A cross-shaped wound was scratched at the bottom of the plate using a 10 µL pipette tip. After gentle rinsing with PBS to eliminate cell debris, the cells were cultured in RPMI-1640 or DMEM medium containing 0.1% FBS. Cell migration was observed and calculated at the indicated times, and the size of the remaining wound was measured using an inverted light microscope.

### Invasion assays

Cells in serum-free conditioned medium were seeded into a BRAND Insert with Matrigel (#BR782806, Sigma-Aldrich). A complete medium containing 10% FBS was added to the lower chamber. After being maintained at 37 °C, cells that did not migrate to the upper chamber were removed, and the invaded cells were fixed with 4% methanol for 30 min and stained with crystal violet. Cells were imaged and counted under a light microscope.

### Colony-formation assays

Cells were seeded in a six-well plate and cultured for two weeks in a complete medium containing 10% FBS. The cells were then fixed with 4% methanol for 30 min and stained with crystal violet (0.5%, #332488, Sigma-Aldrich, St. Louis, MO, USA) for 30 min. The number of colonies was visualized and counted.

### Cell-viability assays

Transfected cells were seeded in 96-well culture plates at a cell density of 500–1000 cells/mL adjusted with 100 μL of the medium. Five wells were plated on the same cells as the replicates. CCK-8 solution was added to 10% at 37 °C for 1 h before any measurements. The absorbance of each cell suspension was measured at 450 nm wavelength using a microplate reader.

### RNA-immunoprecipitation (RIP) assay

A Magna RNA-binding protein immunoprecipitation kit (#17–700, MilliporeSigma, Bedford, MA) was used to determine the relationship between TINCR and DNMT1, according to the manufacturer’s instructions. The antibodies used were negative control normal mouse IgG or human anti-DNMT1. Cell lysates were incubated with RIP buffer containing magnetic beads conjugated with antibody overnight and then incubated with proteinase K to isolate the immunoprecipitated RNA. The co-precipitated RNAs were used for cDNA synthesis and evaluated by qRT-PCR to confirm special binding to DNMT1.

For anti-Ago2 RIP, MDA-MD-231 cells were washed in PBS and lysed in RIP buffer at 4 °C. Cell lysates were treated with magnetic beads conjugated to antibodies against Ago 2 (Millipore) or normal immunoglobulin G (IgG; Millipore), and then were used to perform RIP experiments using an Ago2 antibody as described above.

### Chromatin-immunoprecipitation (ChIP) assay

ChIP assays were performed using a ChIP Assay Kit (#P2078, Beyotime, Shanghai, China), following the manufacturer’s instructions. cells were cross-linked with formaldehyde and sonicated to an average length of 200–1000 bp. The sheared chromatin was immunoprecipitated at 4 °C overnight using an anti-DNMT1 antibody (#GTX116011, GeneTex); IgG (BD Biosciences, San Diego, CA) served as the negative control. The precipitated DNA was amplified by RT-PCR.

### Dual-luciferase reporter assay

Full-length 3ʹ-untranslated regions (UTRs) of human TINCR and USP20 were cloned to generate reporter vectors containing miRNA-binding sites. The 3ʹ-UTRs were then amplified by PCR and cloned into multiple cloning sites in the psi-CHECK-2 luciferase miRNA expression reporter vector. The recombinant plasmids were named pmirGLO-TINCR, pmirGLO-TINCR-mut, pmirGLO-USP20, and pmirGLO-USP20-mut. HEK293T cells were cultured in 24 well plates, and Lipofectamine 2000 (#11668500, Invitrogen) was used to transfect the cells with 20 μmol/L hsa-miR-199a-5p mimic or negative-control (miR NC) mimic and 0.5 mg of the recombinant plasmid. Luciferase activity was detected at 36 h using a dual-luciferase reporter assay kit (#E1910, Promega) and a luminometer (GloMax 20/20, Promega).

### RNA pull-down

In vitro biotin-labeled RNAs were transcribed with 10 × Biotin RNA labeling mix (Roche, cat# 1165597910) and T7 enzyme mix (New England Biolabs, cat# M0251S). Samples were then treated with 10 mM HEPES, 10 mM MgCl2 and 0.1 M NaCl. The RNAs were then incubated with Streptavidin Magnetic Beads (Beyotime Biotechnology, cat# P2151) for 20 min at room temperature with agitation. Protein lysates were then mixed with the RNA-beads complex for 2 h at 4 °C with agitation. The pulldown complexes were then added into loading and boiled at 100 °C for 7 min, followed by western.

### In vivo deubiquitination assay

For cell-based analysis of USP20 deubiquitinating PD-L1 in vivo, 5 × 10^6^ HEK293T cells were individually cotransfected with HA-Ub and Flag-USP20 or Flag expression vectors; another 5 × 10^6^ HEK293T cells were individually cotransfected with HA-Ub, siRNA-USP20, or siRNA-NC. After 40 h of transfection, the cells were treated with 10 μM MG132 for 8 h to accumulate ubiquitinated proteins before being harvested. Cells were then lysed with mild sonication and RIPA buffer (10 mM Tris-HCl [pH 7.4], 150 mM NaCl, 5 mM NaF, 10 mM DTT, 5% glycerol, and 5000 U/mL proteinase inhibitors) containing 2% SDS and boiled at 95 °C for 10 min. The denatured cell lysates were further diluted with SDS-negative lysis buffer, to reduce SDS to 0.2%, and subjected to immunoprecipitation with anti-PD-L1 antibody at 4 °C overnight, followed by western blot analysis with the indicated antibodies. The endogenous ubiquitination levels of PD-L1 were determined using anti-Ub antibody.

### Nuclear/cytoplasmic isolation

Nuclear/cytoplasmic isolation was carried out using NE-PER Nuclear and Cytoplasmic Extraction Reagents (#78835, Thermo Fisher Scientific, USA) according to the manufacturer’s protocol. Cytoplasmic and nuclear fractions were used for RNA extraction. GAPDH and U1 were used as controls for cytoplasmic and nuclear RNAs, respectively.

### Statistical analysis

The expression of each lncRNA was dichotomized using the median expression as the cut-off to define high values (at or above the median) versus low values (below the median). Differences between groups in the in vitro and in vivo experiments were analyzed using a Student’s *t*-test. A Spearman’s correlation analysis was performed to assess the relationship between associated variables. All experiments were performed independently in triplicate. All statistical tests were two-sided, and statistical significance was set at *P* < 0.05.

## Results

### TINCR promotes breast cancer progression in vivo and in vitro

**Fig. 1:**
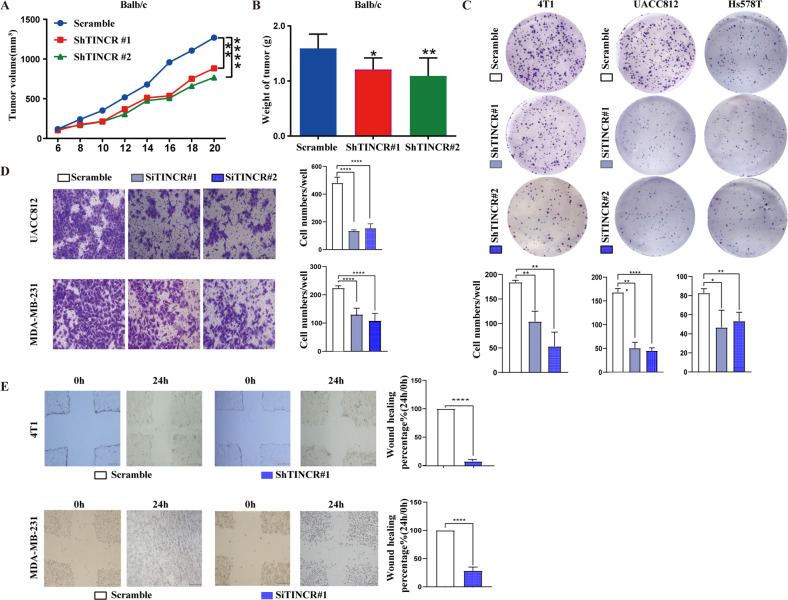
TINCR promotes breast cancer progression in vivo and in vitro. **A**, **B** Tumor growth curves and tumor weights of mice injected with control or knockdown TINCR 4T1 cells. TINCR knockdown inhibited (**C**) colony formation, (**D**) invasiveness, and (**E**) migration of breast cancer cells. Scale bar, 100 μm. Data are presented as means from three independent experiments ± S.D. *P < 0.05, **P < 0.01, ****P < 0.0001.

Three animal cohorts were used to examine the potential effects of TINCR on breast cancer cell proliferation and metastasis in vivo. First, both scrambled and shTINCR 4T1 cells were injected subcutaneously into female Balb/c mice. Compared with the scramble group, tumor growth was significantly inhibited in the TINCR knockdown groups (Figs. 1A and S1A). The tumor weight in the TINCR knockdown group was also lower than that in the control group (Fig. 1B). Moreover, in vitro colony formation assays revealed that depletion of TINCR attenuated cell proliferation (Fig. 1C), and TINCR knockdown decreased invasiveness and rates of migration (Fig. 1D, E).

### TINCR regulates PD-L1 expression in breast cancer

**Fig. 2:**
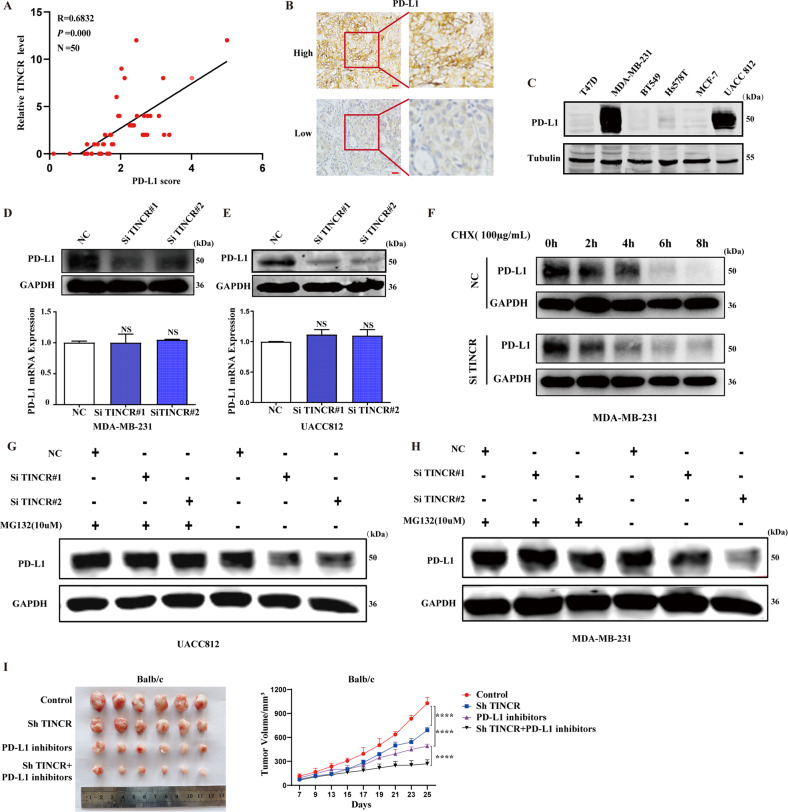
TINCR regulates PD-L1 expression in breast cancer. **A**, **B** Expression of PD-L1 in breast cancer tissues by immunohistochemistry. Scale bar, 400 μm. **C** Basal expression of PD-L1 in different breast cancer cell lines. **D**, **E** Expression of PD-L1 after TINCR knockdown. **F** Expression of PD-L1 was detected after CHX (100 µg/mL) treatment in the control and TINCR knockdown groups. **G**, **H** MDA-MB-231 and UACC812 cells were treated with MG132 to detect the expression of PD-L1 in the control and TINCR-knockdown groups. **I** Tumor tissue and growth curve after the treatment TINCR knockdown, PD-L1 inhibitors and combination therapy. Data are presented as means from three independent experiments ± S.D. ***P < 0.001, ****P < 0.0001.

Among all immune checkpoints, the PD-1/PD-L1 pathway plays an important role in tumor cell immune evasion, making it a potent target in antitumor immunity. LncRNAs and circular RNAs have been shown to regulate the PD-1/PD-L1 pathway, thus affecting immune response and the efficacy of immunotherapy [25–27]. However, TINCR regulation of PD-L1 has not yet been reported. We analyzed 50 breast cancer tissue specimens from HMUCC and found that TINCR was positively correlated with PD-L1 in breast cancer tissues (Fig. 2A, B and Table S4). Breast cancer cell lines with high PD-L1 expression were screened for subsequent experiments (Fig. 2C). We then used siRNA knockdown of TINCR in UACC812 and MDA-MB-231 cells. TINCR knockdown caused a remarkable decrease in the protein level of PD-L1, but had no influence on PD-L1 mRNA (Figs. 2D, E and S1B, C). Therefore, we conclude that TINCR promotes PD-L1 expression at the protein level. Consistent with this finding, the half-life of PD-L1 proteins was markedly shorter in TINCR knockdown cells treated with cycloheximide (CHX) (Fig. 2F). As a common proteasome inhibitor [28], MG132 restored the downregulated PD-L1 levels in cells with TINCR knockdown (Figs. 2G, H and S2). Our results imply that TINCR upregulated the expression of PD-L1 by inhibiting the degradation of PD-L1 proteins. We next assessed the combined effect of TINCR knockdown with PD-L1 inhibitor treatment. In vivo, the combined treatment more effectively inhibited tumor growth than TINCR knockdown did (Fig. 2I). Therefore, TINCR knockdown enhances PD-L1 inhibitor sensitivity in breast cancer, producing a synergistic anticancer effect.

### TINCR recruits USP20 to stabilize PD-L1

**Fig. 3:**
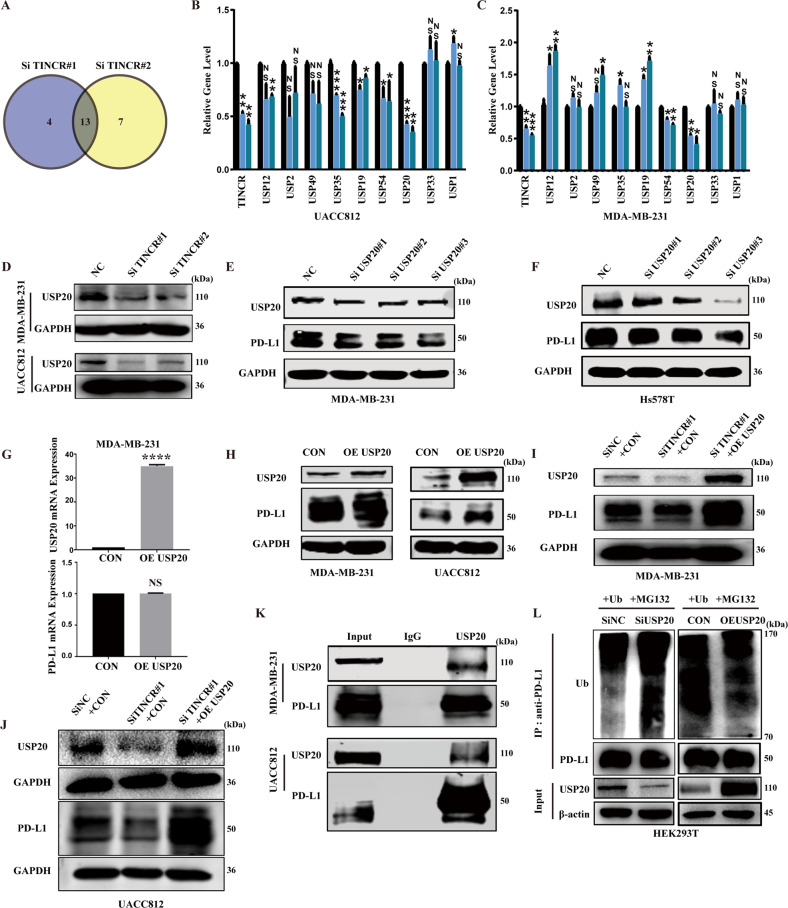
TINCR recruits USP20 to stabilize PD-L1. **A** Transcriptome sequencing in T47D cells after TINCR knockdown. **B**, **C** RNA expression of different deubiquitinases after TINCR knockdown in MDA-MB-231 and UACC812 cells. **D** USP20 protein expression after TINCR knockdown. **E**, **F** PD-L1 protein expression after USP20 knockdown. PD-L1 expression in (**G**) mRNA and (**H**) protein levels after USP20 overexpression in MDA-MB-231 cells. **I**, **J** Detection of PD-L1 expression after TINCR knockdown and USP20 overexpression. **K** CO-IP experiments confirmed that USP20 binds to PD-L1. **L** Ubiquitination assay of PD-L1 in control, USP20-knocked, and USP20-overexpressing HEK293T cells co-transfected with Ub and treated with MG132. Data are presented as means from three independent experiments ± S.D. *P < 0.05, **P < 0.01, ***P < 0.001, ****P < 0.0001.

Given that MG132 prevented the degradation of PD-L1 proteins, we hypothesize that TINCR regulates PD-L1 expression through ubiquitination. Transcriptome sequencing showed that 13 deubiquitinases were reduced following TINCR knockdown (Fig. 3A). By validating the above results in UACC812 and MDA-MB-231 cells, we found that USP20 downregulation was the most pronounced (Fig. 3B, C). Additionally, USP20 protein expression levels decreased after TINCR knockdown (Figs. 3D and S3A–D). After verifying its depletion and efficiency, we found that USP20 knockdown downregulated the expression of PD-L1 (Fig. 3E, F). In addition, USP20 overexpression had no influence on PD-L1 mRNA levels, but upregulated PD-L1 protein levels (Fig. 3G, H). USP20 overexpression rescued the reduction in PD-L1 expression after TINCR knockdown (Fig. 3I, J). Under physiological conditions, USP20–PD-L1 interactions were validated using endogenous immunoprecipitation (Fig. 3K). USP20 depletion induced an increase in ubiquitinated PD-L1 in vivo, which was largely reduced after the rescue of USP20 (Fig. 3L).

### MiR-199a-5p acts as a molecular sponge to mediate the upregulation of USP20 expression by TINCR

**Fig. 4:**
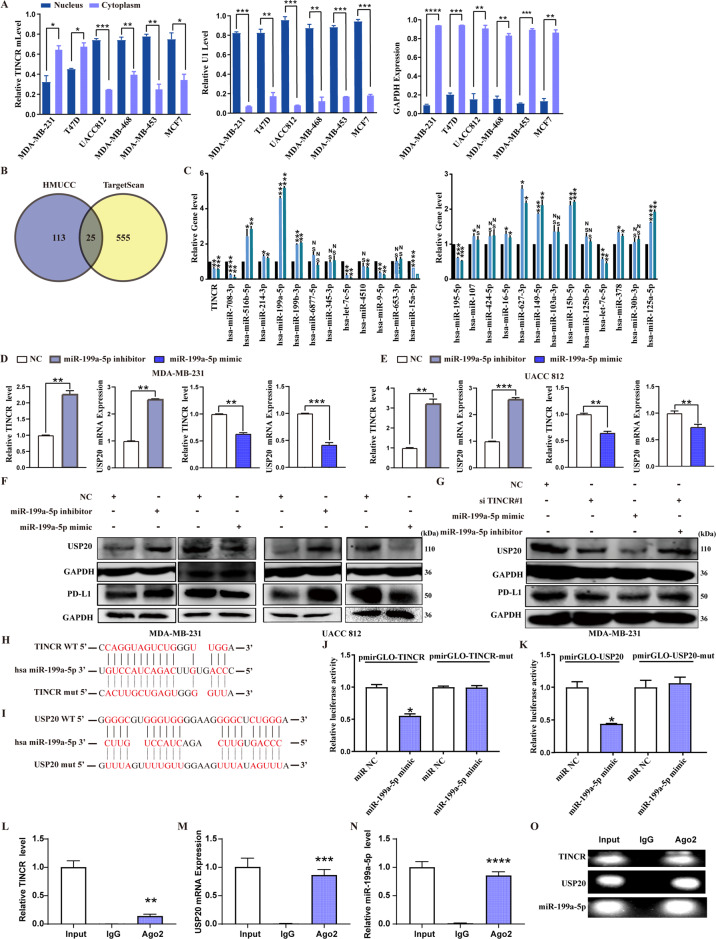
MiR-199a-5p acts as a molecular sponge to mediate the upregulation of USP20 expression by TINCR. **A** Subcellular localization of TINCR in breast cancer cell lines assessed by nuclear/cytoplasmic extract isolation assay. **B** Overlapping miRNAs in transcriptome miRNA sequencing data from the HMUCC cohort and TargetScan. **C** Expression of miRNAs with TINCR knockdown in UACC812 cells. **D**–**G** Expression of TINCR, USP20, and PD-L1. Complementarity between the miR-199a-5p seed sequence and 3ʹ-UTRs of (**H**) TINCR and (**I**) USP20 by using TargetScan and StarBase online databases. Luciferase-reporter assay assessing the interactions between miR-199a-5p and its binding sites or mutated binding sites in 3ʹ-UTRs of (**J**) TINCR and (**K**) USP20 in HEK293T cells. **L**–**O** RNA immunoprecipitation with a monoclonal anti-Ago2 antibody was used to assess endogenous Ago2 binding to RNA; IgG was used as the control. The levels of TINCR, DNMT1, and miR-199a-5p were determined by qRT-PCR and presented as fold enrichment in Ago2 relative to input. Data are presented as means from three independent experiments ± S.D. *P < 0.05, **P < 0.01, ***P < 0.001, ****P < 0.0001.

Considering that the ceRNA mechanism is one way in which lncRNAs participate in the regulation of tumor progression, we assessed whether USP20 can be regulated by TINCR through the ceRNA mechanism. In breast cancer cells, TINCR was localized in both the cytoplasm and nucleus (Fig. 4A). We used the intersection of transcriptome sequencing data from the HMUCC cohort and TargetScan to obtain overlapping miRNAs (Fig. 4B). In UACC812 cells, TINCR knockdown significantly increased the expression of miR-199a-5p (Fig. 4C). MiR-199a-5p was negatively correlated with TINCR and USP20 expression. Knockdown of miR-199a-5p resulted in the upregulation of USP20 and PD-L1 expression (Fig. 4D–F). The downregulation of USP20 and PD-L1 induced by TINCR knockdown was reversed by the miR-199a-5p inhibitor (Fig. 4G). Finally, the dual-luciferase reporter assay showed that both TINCR and USP20 with wild-type 3ʹ-UTRs were regulated by miR-199a-5p, and that this effect could be abolished by mutation of their miRNA-binding sites (Fig. 4H–K). To further verify the interaction between TINCR and miR-199a-5p as well as that between USP20 mRNA and miR-199a-5p, RNA immuno-precipitation experiments were performed in MDA-MB-231 cell extracts using an Ago2 antibody. USP20, TINCR, and miR-199a-5p were enriched in Ago2-containing miRNA containing ribonucleoprotein complexes relative to control IgG immunoprecipitates, suggesting that the Ago2 protein binds directly to USP20, TINCR, and miR-199a-5p in MDA-MB-231 cells (Fig. 4L–O). These results indicated that USP20 and TINCR are direct targets of miR-199a-5p.

### TINCR recruits DNMT1 to the miR-199a-5p locus and suppresses its expression via DNA methylation

**Fig. 5:**
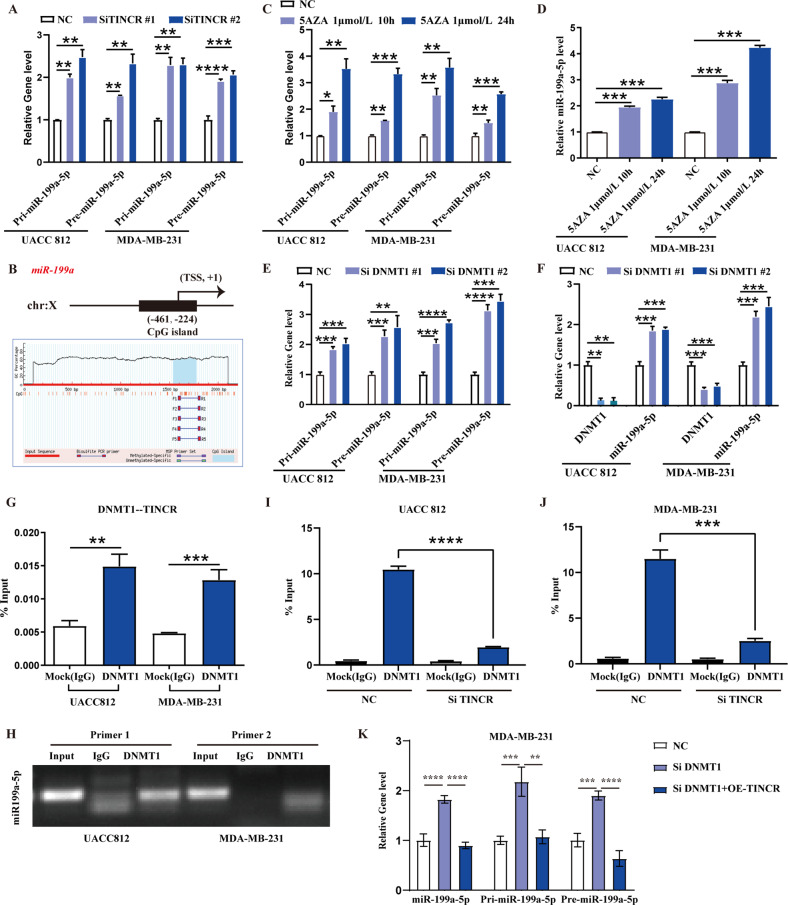
TINCR recruits DNMT1 to miR-199a-5p locus and suppresses its expression via DNA methylation. **A** Expression of pri-miR-199a-5p and pre-miR-199a-5p was detected by qRT-PCR after TINCR knockdown. **B** The predicted CpG island in the miR-199a-5p locus. Expression levels of (**C**) pri-miR-199a-5p, pre-miR-199a-5p and (**D**) miR-199a-5p were detected by qRT-PCR after stimulation with the DNA methyltransferase inhibitor 5AZA. Expression of (**E**) pri-miR-199a-5p, pre-miR-199a-5p and (**F**) miR-199a-5p was detected by qRT-PCR after DNMT1 knockdown. **G** RIP-assay to detect the enrichment of DNMT1 on TINCR. IgG: negative control. **H**–**J** ChIP assay to detect the enrichment of DNMT1 at the promoter of the miR-199a-5p locus. **K** The expression of miR-199a-5p, pri-miR-199a-5p and pre-miR-199a-5p was detected by qPCR after DNMT1 knockdown and TINCR overexpression. Data are presented as means from three independent experiments ± S.D. *P < 0.05, **P < 0.01, ***P < 0.001, ****P < 0.0001.

As mentioned above, TINCR was localized to the nucleus in addition to the cytoplasm and TINCR knockdown upregulated the expression of miR-199a-5p (Fig. 4C). We hypothesize that TINCR in the nucleus may exert its regulatory effect through epigenetic modification of the miR-199a-5p locus. The expression of pri-miR-199a-5p and pre-miR-199a-5p increased after TINCR knockdown in UACC812 and MDA-MB-231 cells (Fig. 5A). Because DNA methylation is one of the major epigenetic modifications that downregulates the expression of miRNA [29], we investigated whether TINCR can mediate the methylation of the miR-199a-5p locus. We observed enrichment peaks for DNMT1 in the promoter region of miR-199a in the ENCODE database (Fig. S4). We obtained information for the transcriptional start site of miR-199a-5p from the University of California at Santa Cruz (UCSC) database (http://genome.ucsc.edu/cgi-bin/hgGateway) and located a CpG island in the promoter region of the miR-199a-5p locus from public data available at Li Lab (http://www.urogene.org/methprimer/index.html) (Fig. 5B). The expression of pri-miR-199a-5p, pre-miR-199a-5p, and miR-199a-5p was significantly upregulated after treatment with the DNA methyltransferase inhibitor, decitabine (5AZA), in UACC812 and MDA-MB-231 cells (Fig. 5C, D). Since DNMT1 is a key gene for DNA methylation in epigenetic modifications of the mammalian genome, we assessed whether DNMT1 regulates miR-199a-5p expression. As expected, the expression of pri-miR-199a-5p, pre-miR-199a-5p, and miR-199a-5p was significantly upregulated after DNMT1 knockdown in UACC812 and MDA-MB-231 cells (Fig. 5E, F). The RIP results showed that DNMT1 was significantly enriched in TINCR (Figs. 5G and S5). ChIP-PCR showed that DNMT1 was significantly enriched in the promoter region of miR-199a-5p (Fig. 5H), which was significantly reduced after TINCR knockdown (Fig. 5I, J). Moreover, RNA pull-down confirmed the direct combination of TINCR and DNMT1 (Fig. S6 and Table S5). To verify the interaction among the TINCR, DNMT1, and miR-199a-5p, we performed the rescue assay, the results showed that TINCR recruits DNMT1 to regulate miR-199a-5p expression (Figs. 5k and S7).

### IFN-γ promotes TINCR expression by upregulating STAT1

**Fig. 6:**
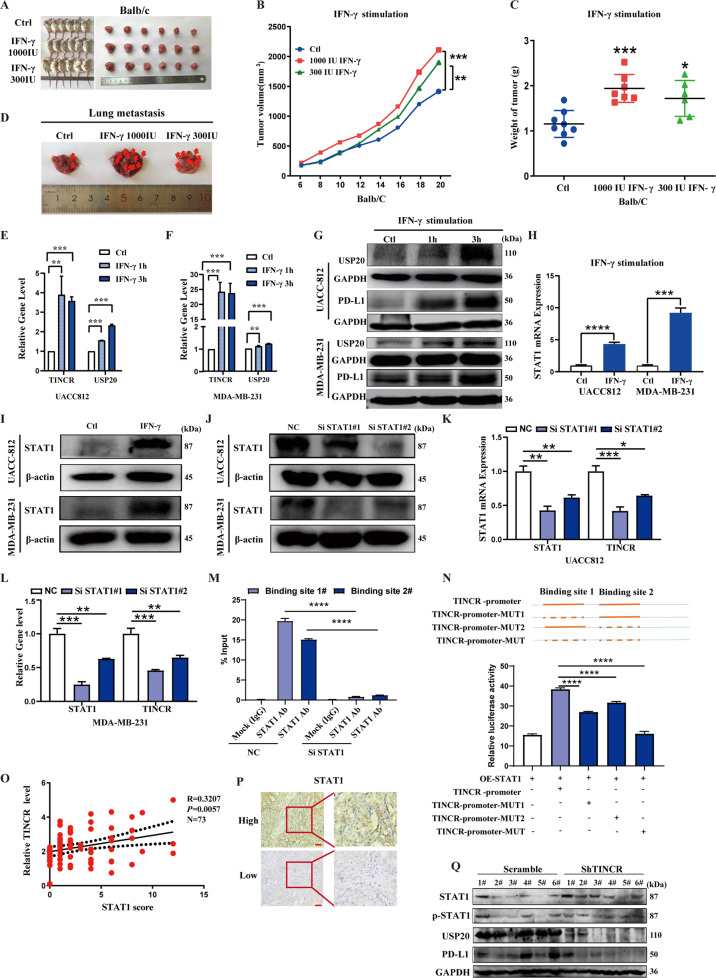
IFN-γ promotes TINCR expression by upregulating STAT1. **A** Tumorigenesis and tumor tissues in each group of Balb/c mice (*n* = 6/group). **B**–**D** Tumor growth, weight, and number of nodules in the lungs of Balb/c mice per group. **E**–**I** Expression of STAT1, TINCR, USP20, and PD-L1, after IFN-γ stimulation. **J**–**L** TINCR and STAT1 were detected using qRT-PCR and western after STAT1 knockdown. **M**, **N** ChIP and luciferase-reporter assay were used to verify the interaction between TINCR and STAT1. **O**, **P** Expression of STAT1 in breast cancer tissues by immunohistochemistry; scale bar, 400 μm. **Q** Expression of STAT1, p-STAT1, USP20, and PD-L1 in mouse tissues of the scramble and TINCR knockdown groups (*n* = 6/group). Data are presented as means from three independent experiments ± S.D. *P < 0.05, **P < 0.01, ***P < 0.001, ****P < 0.0001.

To further elucidate the molecular mechanism, we investigated the upstream regulatory mechanism of TINCR. IFN-γ is a pleiotropic cytokine with antiviral, antitumor, and immunomodulatory effects that play an important role in coordinating innate and acquired immunity [30]. In the tumor microenvironment, IFN-γ consistently mediates tumorigenic and antitumor immunity. On the one hand, it exerts antitumor effects by mediating apoptosis of cancer cells [31], inhibiting tumor angiogenesis [32], stimulating polarization of M1 macrophages, and inhibiting their M2 phenotype [33]. On the other hand, it can induce epithelial-to-mesenchymal transition processes to promote tumor metastasis [34] and impair T cell immune responses, leading to immune escape [35]. Moreover, previous studies have shown that IFN-γ is a key driver of PD-L1 expression and host tumor-infiltrating lymphocytes, which mediate PD-L1 expression through IFN-γ secretion in many cancer types and participate in the regulation of tumor immune escape [36–38]. We confirmed in vitro phenotypic experiments that IFN-γ can promote the proliferation, invasion, and metastasis of breast cancer cells (Fig. S8). Based on these results, we reasoned that IFN-γ might contribute to PD-L1-induced immune escape by affecting the expression of TINCR. To confirm its immunosuppressive effects, we used IFN-γ at different concentrations (300 or 1000 IU) in a subcutaneous tumor model (Balb/c mice injected with 4T1 cells in vivo), we found that IFN-γ stimulation significantly promoted tumor growth compared to the control group (Fig. 6A, B). The tumor weights of each group are shown in Fig. 6C. Compared with the vehicle-treated group, the administration of IFN-γ increased the number of tumor nodules in the lungs (Fig. 6D). Additionally, IFN-γ stimulation increased TINCR, USP20, and PD-L1 mRNA and protein expression levels relative to the control and reduced miR-199a-5p expression in UACC812 and MDA-MB-231 cells (Figs. 6E–G and S9).

To investigate the transcriptional regulation of IFN-γ and TINCR, we used JASPAR (http://jaspar.genereg.net/) to search for transcription factors that might be enriched at the promoter of the TINCR locus. This analysis yielded putative STAT1-binding sites in the region upstream of the TSS of TINCR (Fig. S10A, B). Moreover, there were obvious enrichment peaks for STAT1 in the promoter region of TINCR in the ENCODE database (Fig. S10C). As predicted, IFN-γ stimulation led to an increase in STAT1 relative to its levels in control cells (Fig. 6H–I). We used qRT-PCR to examine the effect of STAT1 knockdown on TINCR expression in breast cancer cells. The expression level of TINCR was also downregulated in UACC812 and MDA-MB-231 cells after STAT1 knockdown (Fig. 6J–L). The dual-luciferase reporter and ChIP assay showed that STAT1 binds directly to the TINCR promoter (Fig. 6M, N and Tables 6–7. In the immunohistochemical analysis of the HMUCC cohort, we found that STAT1 level was higher in tissues overexpressing TINCR than in underexpressed tissues (Figs. 6O, P and Table S8). In the subcutaneous tumor model, downstream signaling molecules were downregulated in the TINCR knockdown group relative to the control group, and there were no significant changes in STAT1 and p-STAT1, and the protein levels of PD-L1 and USP20 were also downregulated (Fig. 6Q). Our findings suggest that IFN-γ promotes tumor immune escape by transcriptionally activating TINCR via STAT1, which in turn upregulates PD-L1 expression.

## Discussion

**Fig. 7:**
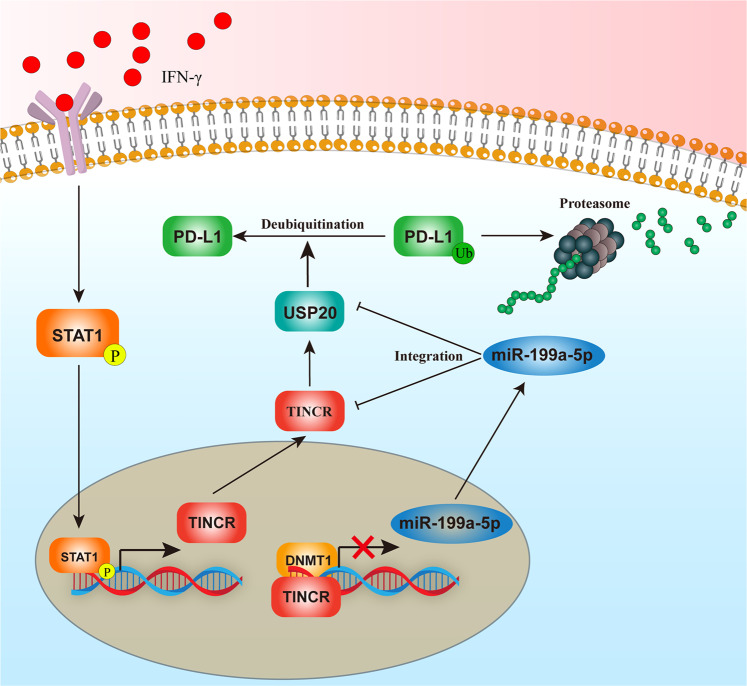
Molecular mechanism of TINCR–USP20–PD-L1 and their upstream regulation. IFN-γ upregulates the expression of TINCR by promoting the expression of STAT1, and the downstream genes, include USP20 and PD-L1, of TINCR via downregulating miR-199a-5p.

Breast cancer has become one of the greatest threats to women’s health worldwide. The clinical application of immunotherapy with PD-L1 inhibitors has greatly improved the prognosis of breast cancer patients [39]. However, owing to the low response rate of PD-L1 inhibitors in breast cancer patients, most are combined with chemotherapy or other drugs to achieve the desired therapeutic effect [40]. Therefore, there is an urgent need to explore the mechanism of abnormal PD-L1 expression in breast cancer and to improve the treatment response rates of PD-L1 inhibitors. LncRNAs participate in the regulation of PD-L1 in a variety of malignant tumors and promote immune escape in tumor cells [41]; however, there are few reports on the regulation of PD-L1 expression by lncRNAs in breast cancer. In this study, we revealed that the lncRNA TINCR upregulates USP20 expression through the dual mechanism of ceRNA interaction and miR-199a-5p transcription inhibition; this in turn leads to abnormally high expression levels of PD-L1 by inhibiting its ubiquitination. We also revealed that, as part of the upstream regulatory mechanism, the INF-γ–STAT1 signaling axis promoted TINCR transcription in breast cancer (Fig. 7).

In tumors, lncRNAs participate in different regulatory mechanisms depending on their subcellular localization. LncRNAs in the nucleus can interact with DNA, epigenetic modification complexes, or transcription factors to regulate the expression of oncogenes or tumor suppressor genes. LncRNAs in the cytoplasm are mainly involved in the regulation of the post-transcriptional level of genes, such as the regulation of mRNA or protein stability through the ceRNA mechanism and the regulation of protein translation [42, 43]. In this study, we found that TINCR was expressed in both the cytoplasm and nucleus of breast cancer cells. In the cytoplasm, we found that TINCR acts as a molecular sponge for miR-199a-5p and upregulates the stability of USP20 mRNA through the ceRNA regulatory mechanism, thus promoting the expression of PD-L1 by inhibiting its ubiquitination. In the nucleus, TINCR could recruit DNMT1 to promote mtethylation and, consequently, inhibit the transcription of miR-199a-5p. The decrease in miR-199a-5p weakens its inhibition of USP20 mRNA stability in the cytoplasm, which eventually leads to the upregulation of PD-L1 expression. In examining the upstream regulatory mechanism of TINCR in breast cancer, we found that IFN-γ stimulation can activate downstream STAT1 signaling and that STAT1 migrates to the nucleus to promote the transcription of TINCR, thus regulating the expression of downstream miR-199a-5p, USP20, and PD-L1. However, after three hours of IFN-γ treatment, miR-199a-5p increased slightly. We consider that this may be due to short IFN-γ stimulation time and unstable expression. Then we extended the processing time and confirmed that IFN-γ Stimulation can indeed downregulate the expression of miR-199a-5p.

This is the first study to clarify the mechanism by which TINCR upregulates USP20 and PD-L1 through the dual role of ceRNA interaction and miR-199a-5p transcription inhibition. Additionally, we verified the specific molecular mechanism of INF-γ–TINCR–USP20–PD-L1 in upregulating PD-L1 expression, thereby inducing breast cancer immune escape and promoting disease progression. Based on this mechanism, we emphasize the potential of TINCR as a target for breast cancer immunotherapy, specifically by combining TINCR knockdown with a PD-L1 inhibitor.

## Supplementary information

supplementary figure 1-5

supplementary figure 6-8

supplementary figure 9-10

table S1

table S2

table S3

table S4

table S5

table S6

table S7

table S8

Supplementary figure and table legends

Revised-western blot original data

Reproducibility Checklist

## Data Availability

All data generated or analyzed during this study are included in this published article and its Additional files. Additional data are available from the corresponding author on reasonable request.
