# Supplementary figures and images for: LncRNA TINCR impairs the efficacy of immunotherapy against breast cancer by recruiting DNMT1 and downregulating MiR-199a-5p via the STAT1–TINCR-USP20-PD-L1 axis
Source: Cell Death Dis. 2023 Feb 1;14(2):76. doi: 10.1038/s41419-023-05609-2 (PMC9892521; doi:10.1038/s41419-023-05609-2)

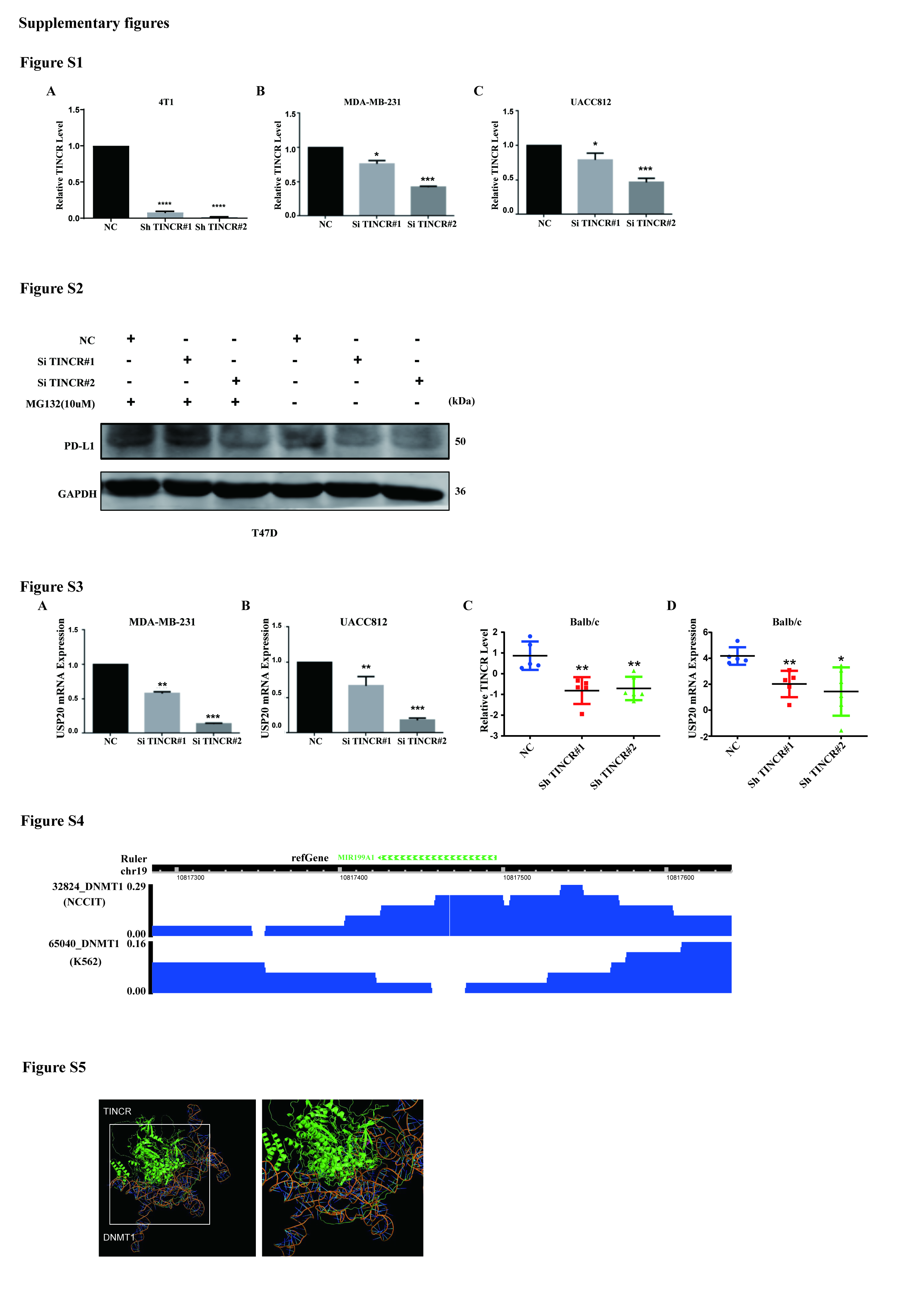

Supplement: Supplementary file 1 — supplementary figure 1-5 [file 41419_2023_5609_MOESM1_ESM.tif]

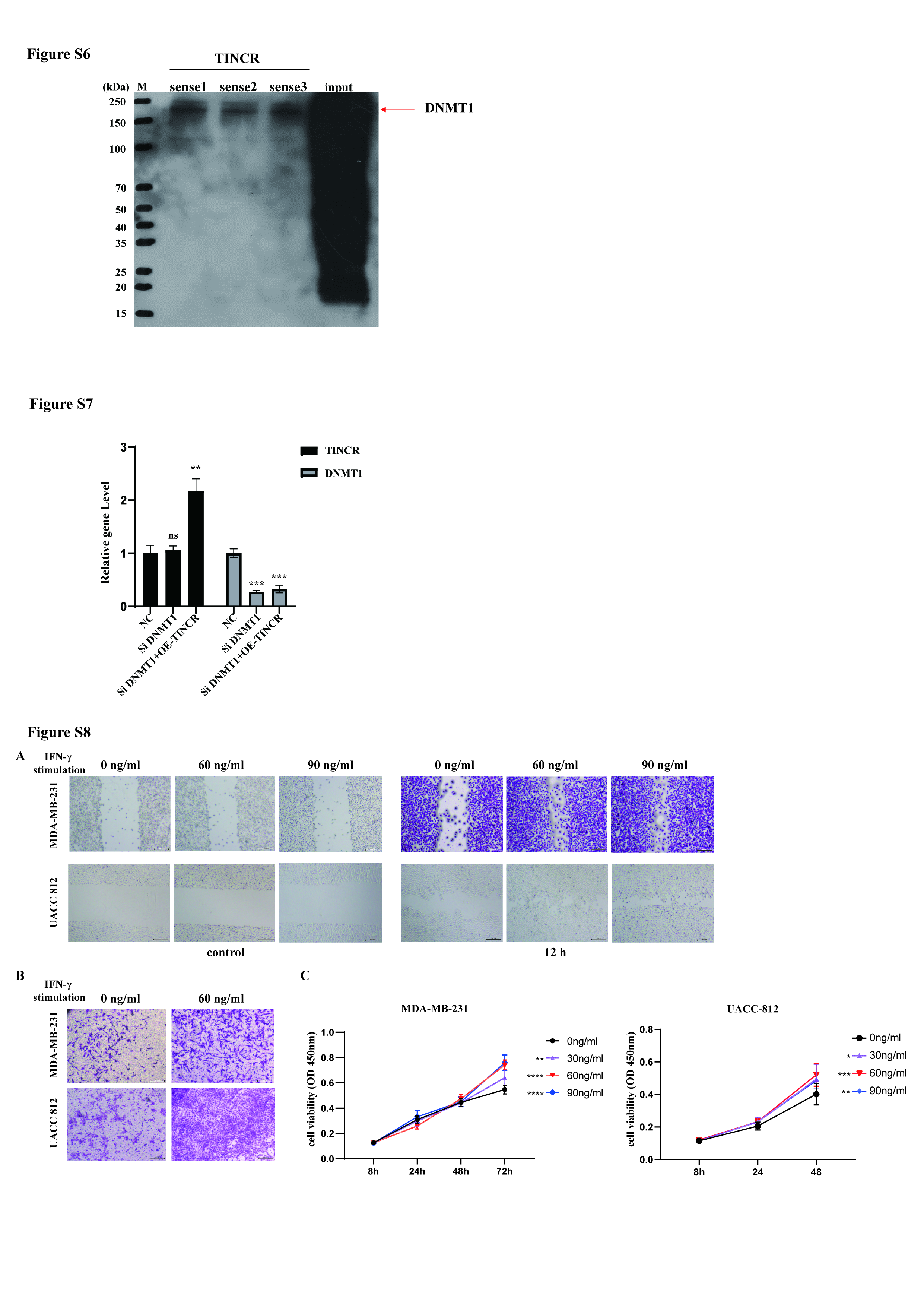

Supplement: Supplementary file 2 — supplementary figure 6-8 [file 41419_2023_5609_MOESM2_ESM.tif]

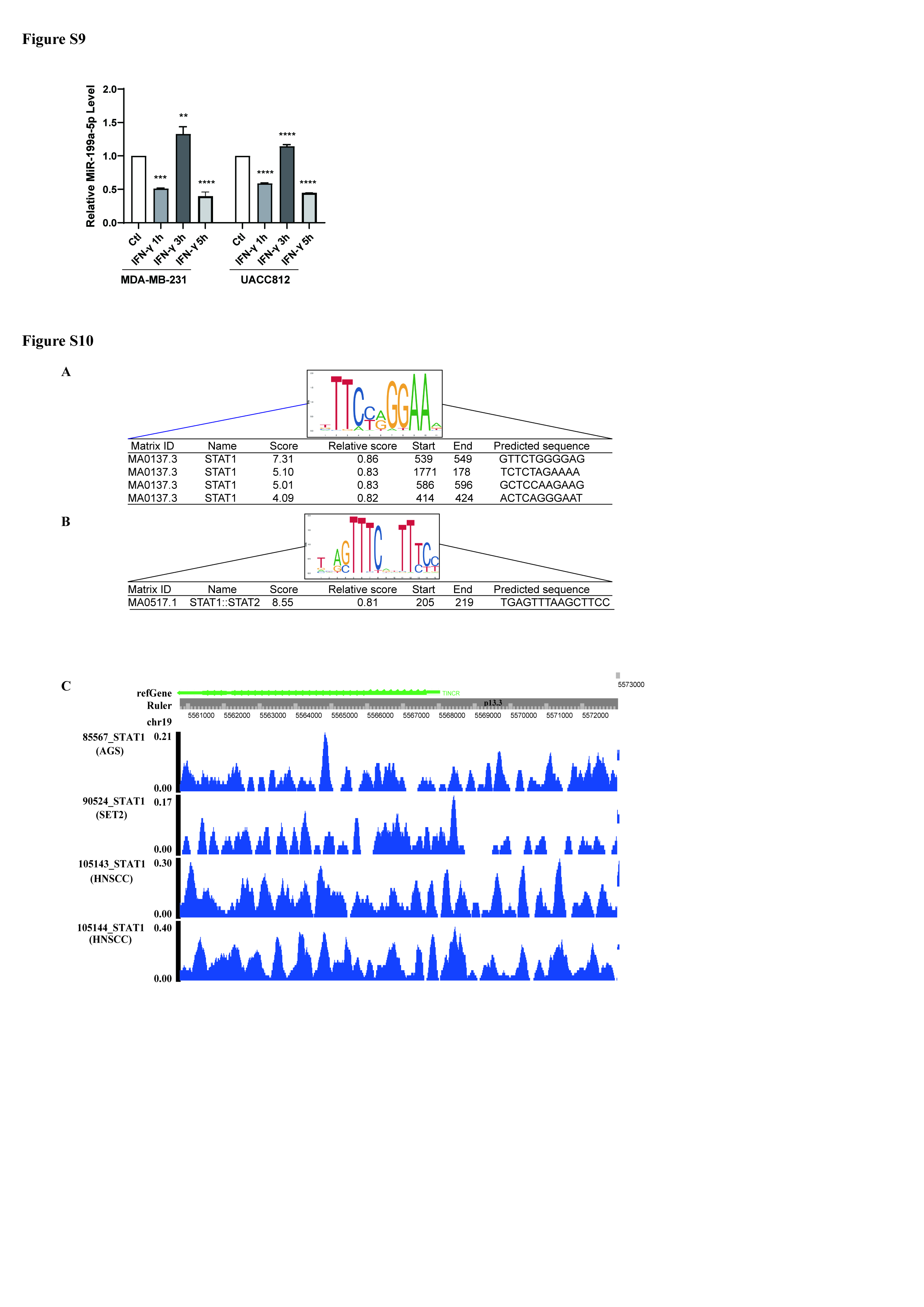

Supplement: Supplementary file 3 — supplementary figure 9-10 [file 41419_2023_5609_MOESM3_ESM.tif]
