# Supplementary material for: LncRNA TINCR impairs the efficacy of immunotherapy against breast cancer by recruiting DNMT1 and downregulating MiR-199a-5p via the STAT1–TINCR-USP20-PD-L1 axis
Source: Cell Death Dis. 2023 Feb 1;14(2):76. doi: 10.1038/s41419-023-05609-2 (PMC9892521; doi:10.1038/s41419-023-05609-2)
Supplement: Supplementary file 4 — table S1 [file 41419_2023_5609_MOESM4_ESM.docx]

| siTINCR-1 | GCAUGAAGUAGCAGGUAUUUU |
| --- | --- |
| siTINCR-2 | GAUCCCGAGUGAGUCAGAAUU |
|  |  |
| siUSP20-1 | AGCACAACTTGACCGTGAA |
| siUSP20-2 | GTCACCGGACCAAACCTAT |
|  |  |
| shTINCR-1(mouse) | CCACATCAAGGTACACCTA |
| shTINCR-2(mouse) | AAGCCACAATGTGGAGAGCAT |
|  |  |
| siDNMT-1 | GAAGAGACGTAGAGTTACA |
| siDNMT-2 | GGAACTTTGTCTCCTTCAA |
|  |  |
| siSTAT1-1 | CTGGATATATCAAGACTGA |
| siSTAT1-2 | GCACGCTGCCAATGATGTT |
|  |  |
| miR-199a-5p-mimic | CCCAGUGUUCAGACUACCUGUUC ACAGGUAGUCUGAACACUGGGUU |
| miR-199a-5p-inhibitor | GAACAGGUAGUCUGAACACUGGG |

**Table. S1 The sequences of siRNA and miR-199a-5p mimic or inhibitor.**
