# Supplementary material for: LncRNA TINCR impairs the efficacy of immunotherapy against breast cancer by recruiting DNMT1 and downregulating MiR-199a-5p via the STAT1–TINCR-USP20-PD-L1 axis
Source: Cell Death Dis. 2023 Feb 1;14(2):76. doi: 10.1038/s41419-023-05609-2 (PMC9892521; doi:10.1038/s41419-023-05609-2)
Supplement: Supplementary file 5 — table S2 [file 41419_2023_5609_MOESM5_ESM.docx]

**Table. S2 The information of plasmids.**

1、TINCR(NR_027064)-human

Vector name：GV658

Component sequence：CMV enhancer-MCS-polyA-EF1A-zsGreen-sv40-puromycin

Cloning site：KpnI / PacI

Plasmid Sequence: TCCCAGGTCCAACTGCACCTCGGTTCTTAATACGACTCACTATAGGGCTAGCGTTTAAACGGGCCCTCTAGACTCGAGCGGCCGCCACTGTGCTGGATATCTGCAGAATTCCACCACACTGGACTAGTGGATCCGAGCTCGGTACCAGAGCTGGAGCCGGAGCCGGGCGGGCGCCATGGAGGGGCTGCGGCGGGGGCTGTCGCGCTGGAAGCGCTACCACATCAAGGTGCACCTGGCGGACGAGGCGCTGCTGCTACCGCTGACCGTGCGGCCGCGGGACACGCTCAGCGACCTGCGCGCCCAGCTGGTGGGCCAGGGCGTGAGCTCCTGGAAGCGCGCCTTCTACTACAACGCGCGGCGGCTGGACGACCACCAGACGGTGCGCGACGCGCGCCTGCAGGACGGCTCGGTGCTGCTGCTCGTCAGCGACCCCAGGTAGTCTGGGTTGGAGGAGGCAGAGCCATGACCAAGGGGACCTGGGTACTGGCTGAAGGAATAGGCTGGGGTAGAGGGCACTTTTGGAAGGCACTTCTCCTGCCTCCCGGGAGCCTAGATCTCACTCCAGGGTCTGGGCTCCCAGGTGGACCATGAAACCCTGGCCTGACCAGCTGATGCACACTGCTTCAGACACTCCTGCTGGAGCCCCAGTCCCTGACAAGGACCTAGGACATTTTTGCTCCTGCCCAGCCTATCGGGAGGGAGCCTTGAGCCTTTCAGCTCTGCTGTGTGACTTTGAGGTTGTTGCTCCCCTCTTGGGGCCCTGGGTGCCCTGTCTTCAGTGGAAAGCACTGTGCCACCTTGGAAAGCTCCCATGGGCAGCCAGAGGGCATCGCAAGAAGAGAAGCACAGAAGGGGCAGGAGAGACACTCAGAGGCACTTCCGCTCTTGCCCAGGACATTTTCCCAGCCACACCTTTGCCCAAGCCGTGCCCCCTGCCTGGAGCACTTTTCAACCTCTTCTCTGCAGCTCCAATACACCTGGGATTGCAGTCTCCTCCAGGAAGTCTTCTCAGATTCCCTCCTTCCCAGCCAGAGAGCACCTAGCCTTCTTTGGGGCCCCCACAGGCCCTTTGTGCAGTGAACAGCCCTGGCTGGGGGTGCAGCCAGTCGTGTCCGAACTCTCCAATGACTAAGCGGGGAGATGCGGACATCCTAGCTCCTTCTCAGGCCTCCAACTGTGCCCCATTTCCACCCCCAAATACCTCCCCAGGAGGCACCTGTGCCCACCCCCTGGGCTGTTTCCCCCTTCCCCTTAAATCCGGATGCCTCGTCTTGCATAGGGTCTTGGGGCAGCGGGGAAGGGGTTCTGAAGAACTCTGGCCAAGAGGACGAGGATCTGGGGGTGGGGAACTGGGCCTAGCTGTGGGAGGTCATCGCGGGGCATTGCAGGGAGTGCGTTGTGGGAATTCCGGGTGGAGACCCTCAGGGCAGTGTGCCGGGCCTCCGTTGCACCTCTGACCTGCAGCCACGTCATCGTTGCGCAGCCCCTGGGGAGGGTCTTTGGGGAGGGGGTGCCGAGGCTACGCATGTCCTCGAGGGCCGTGTACACTCTCCAGGCACCATGGGCGGAGGCGCCAGAGGCTGGGAAGAAATAATGTTTTAGTTAAGAGTCCTGTTGGCTGCAGGACTCAGAGCATGGACAGGTGGATAGTAAATCACCACCACGGGGACAGCCGTGCCCAGACTGTGCGTTTGCTTAGCTCGGGGACAGCACTTGGCCCGGGGTCTCCTGCTCGCCTCCCTTCAGAGCATCTGCCAAACTTCGGGCATCTACCCTGCAATTCCCGCTTGGCTGAGAGGAGGGGGAGAAGGAGGGGAAGAGAGAGGAGCCCCACTTCACTCCGGCAGGCAGACCTGCTGGAGCTGCTTTGCAGAATGACTTGGGTCTTGCTGGCCCCTGGGTGTGCCTGGAGGGGGGTCTTCCTATCCCCCTCCACCTCTCCCCTTGGTGCCTAACCCAGGACTTTGTCCCCAGAGACCCACTGTGTGCCCCCTGGAGCTCCTCCTAGAGCAGAGTCTGCTGCTGTCTGTTCTGCAGAGGGTAGCCTGAGGCCCAAGGAGGTTGTCAGGGACACACAGCAGGGGGAGGCAGCCCAGATCCCTGCTCATGCGTGGTTTGGGCCTCTGCTAACAGCTGGGCCACATGCGTGTATCTAGAGCCTGGGTCCCCTCTGTCCTCATTTCCCTGTGCATATTCAGGGACATGCCTCGTCAGGGTTTGGCAAGAAAATGGGGCATTTAATAATCTCCCCAGTTTCATTCCTTGGTCCTTACTCCATGCCAGGACTTGTGCACATCTTTTTGGAGCCCTGTCTCACTTGATTGACAGCCGGCCAGGCGCGGTGGCTCACGCCTGTAATCCCAGCACTTTGGGAGGACGAGGCAGGCGGATCATGAGGTCAGGAGATGGAGACCATCCTGGCTAACACGGTGAAACCCCGTCTCTACTAAAAGTACAAAAAAAATAGCCGGGCCTGGTGGCGGGCGCCTGTAGTCCCAGCTACTTGGTAGGCTGAGGCAGGAGAATGGCGGGAACCCGGGAGGCAGAGGTTGCAGTGAGCTGAGATCACGCCACTGCACTCCAGCCTGGGCGACAGAGCGAGACTCCGTCTCAAAAAAAAAAAAAAAAAAGATTGACAGCCTCACTGCTCAGGGGCCAGGATAACAGCCCATTTTACAGATGGCTTTTGGCTTGCCCAAGGTCACCACCCTCTGAACTGAGGCGTCCCCAACCCATGCCGGGAGCTGGTGGAGGAAGTGGGGAAGGGTTTCCCGCTTCTCCACTGGGGGTGGAGGTGGGAGAAAGCATTTTCAGGGTGTTTTGACATTTAACAAACAGTATGCAAATCACATGCAAATCGCATGCAAATCACCACCCATTTCTTAAGCCTCGACAGCGCTGGCATGTTCTGAAATAATAATGTTGGACCTGTCCCTTGATGATGACCTGGGTCGGGAGGTGCACGGCCTTTTCCTTCCTCCGTCTTCCCTTCCCTTCCCTTCCCTTCCCATCTGTTCTCCCTTCCTTCGCCCCCTTCCTTTCCTCTCCCTCCCTATCTTCCATTTTTCTCTTCTTCCTTCACATCATTTGTCCAAGACCTCCCTGGCCTGAGATAAACATAAGTTGCAAAAGTGGCTTCCTGCAGCCCATTTTCCAGGTTGGGAAACTGAGGCATGGGCCTAAGGTCCCACAGCCACACCGAGGAAAGCAGCCCCCACTGAATGTCACCATGCACCAAAGTGCATGGTCCTCGTGACACAAAGAGGGGAGATGACAGTGGCTGGAGTTGTCAGAGCTGCTTTGAGGAAGCTCCAGGCCTGATAGGCTTGACAGGGCCAAGGGGAACTATTGTGGAATGTCTTGGCCTTGAATGACAGGCTGCTGATTTTGGATCTGGAGAAAGCTCACACTGACTCTTCCTGCTCCCCCAGTCTGTTTTTCTCCTGCTACTCCATCCATGTATCCCTGAGTTTGGGCCACAGTCCCTCCACTTGGTTTGCATGTCCCACCTTCTTTGTTTGGCAAGCTCCTATTCATCCTTCAAAGCCCTAGCTTCAATACCTGCTACTTCATGCAGCCATTGCTTATCCTTCAAGGAGAGCCTACTTCCCTCAAGGACCCTGCTGCTTCCAGTCTCACCTTCTGACTCACTCGGGATCCACTGAACTGGGAGGTCTGTGTCTCCTCCCAGCAGAGTCATCACTACCTTTGGGGCCGCAGGATCACCCAGCTTGGAACTAGATACAGAAATGCTGTTTTGAGAGTGTACTGAATAAAAGATTACATGTTTGAAAACAATTAATTAAACCGGTAATAAAATATCTTTATTTTCATTACATCTGTGTGTTGGTTTTTTGTGTGAATCGATAGTACTAACATACGCTCTCCATCAAAACAAAACGAAACAAAACAAACTAGCAAAATAGGCTGTCCCCAGTGCAAGTGCAGGTGCCAGAACA

2、STAT1(NM_007315)-human

Vector name：GV657

Component sequence：

CMV enhancer-MCS-3flag-polyA-EF1A-zsGreen-sv40-puromycin

Cloning site：BamHI / KpnI

Plasmid Sequence:

CTGCACCTCGGTTCTTAATACGACTCACTATAGGGCTAGCGTTTAAACGGGCCCTCTAGACTCGAGCGGCCGCCACTGTGCTGGATATCTGCAGAATTCCACCACACTGGACTAGTGGATCCCGCCACCATGTCTCAGTGGTACGAACTTCAGCAGCTTGACTCAAAATTCCTGGAGCAGGTTCACCAGCTTTATGATGACAGTTTTCCCATGGAAATCAGACAGTACCTGGCACAGTGGTTAGAAAAGCAAGACTGGGAGCACGCTGCCAATGATGTTTCATTTGCCACCATCCGTTTTCATGACCTCCTGTCACAGCTGGATGATCAATATAGTCGCTTTTCTTTGGAGAATAACTTCTTGCTACAGCATAACATAAGGAAAAGCAAGCGTAATCTTCAGGATAATTTTCAGGAAGACCCAATCCAGATGTCTATGATCATTTACAGCTGTCTGAAGGAAGAAAGGAAAATTCTGGAAAACGCCCAGAGATTTAATCAGGCTCAGTCGGGGAATATTCAGAGCACAGTGATGTTAGACAAACAGAAAGAGCTTGACAGTAAAGTCAGAAATGTGAAGGACAAGGTTATGTGTATAGAGCATGAAATCAAGAGCCTGGAAGATTTACAAGATGAATATGACTTCAAATGCAAAACCTTGCAGAACAGAGAACACGAGACCAATGGTGTGGCAAAGAGTGATCAGAAACAAGAACAGCTGTTACTCAAGAAGATGTATTTAATGCTTGACAATAAGAGAAAGGAAGTAGTTCACAAAATAATAGAGTTGCTGAATGTCACTGAACTTACCCAGAATGCCCTGATTAATGATGAACTAGTGGAGTGGAAGCGGAGACAGCAGAGCGCCTGTATTGGGGGGCCGCCCAATGCTTGCTTGGATCAGCTGCAGAACTGGTTCACTATAGTTGCGGAGAGTCTGCAGCAAGTTCGGCAGCAGCTTAAAAAGTTGGAGGAATTGGAACAGAAATACACCTACGAACATGACCCTATCACAAAAAACAAACAAGTGTTATGGGACCGCACCTTCAGTCTTTTCCAGCAGCTCATTCAGAGCTCGTTTGTGGTGGAAAGACAGCCCTGCATGCCAACGCACCCTCAGAGGCCGCTGGTCTTGAAGACAGGGGTCCAGTTCACTGTGAAGTTGAGACTGTTGGTGAAATTGCAAGAGCTGAATTATAATTTGAAAGTCAAAGTCTTATTTGATAAAGATGTGAATGAGAGAAATACAGTAAAAGGATTTAGGAAGTTCAACATTTTGGGCACGCACACAAAAGTGATGAACATGGAGGAGTCCACCAATGGCAGTCTGGCGGCTGAATTTCGGCACCTGCAATTGAAAGAACAGAAAAATGCTGGCACCAGAACGAATGAGGGTCCTCTCATCGTTACTGAAGAGCTTCACTCCCTTAGTTTTGAAACCCAATTGTGCCAGCCTGGTTTGGTAATTGACCTCGAGACGACCTCTCTGCCCGTTGTGGTGATCTCCAACGTCAGCCAGCTCCCGAGCGGTTGGGCCTCCATCCTTTGGTACAACATGCTGGTGGCGGAACCCAGGAATCTGTCCTTCTTCCTGACTCCACCATGTGCACGATGGGCTCAGCTTTCAGAAGTGCTGAGTTGGCAGTTTTCTTCTGTCACCAAAAGAGGTCTCAATGTGGACCAGCTGAACATGTTGGGAGAGAAGCTTCTTGGTCCTAACGCCAGCCCCGATGGTCTCATTCCGTGGACGAGGTTTTGTAAGGAAAATATAAATGATAAAAATTTTCCCTTCTGGCTTTGGATTGAAAGCATCCTAGAACTCATTAAAAAACACCTGCTCCCTCTCTGGAATGATGGGTGCATCATGGGCTTCATCAGCAAGGAGCGAGAGCGTGCCCTGTTGAAGGACCAGCAGCCGGGGACCTTCCTGCTGCGGTTCAGTGAGAGCTCCCGGGAAGGGGCCATCACATTCACATGGGTGGAGCGGTCCCAGAACGGAGGCGAACCTGACTTCCATGCGGTTGAACCCTACACGAAGAAAGAACTTTCTGCTGTTACTTTCCCTGACATCATTCGCAATTACAAAGTCATGGCTGCTGAGAATATTCCTGAGAATCCCCTGAAGTATCTGTATCCAAATATTGACAAAGACCATGCCTTTGGAAAGTATTACTCCAGGCCAAAGGAAGCACCAGAGCCAATGGAACTTGATGGCCCTAAAGGAACTGGATATATCAAGACTGAGTTGATTTCTGTGTCTGAAGTTCACCCTTCTAGACTTCAGACCACAGACAACCTGCTCCCCATGTCTCCTGAGGAGTTTGACGAGGTGTCTCGGATAGTGGGCTCTGTAGAATTCGACAGTATGATGAACACAGTAGTACCAAGCTTAAGTGACTACAAGGATGACGATGACAAGGATTACAAAGACGACGATGATAAGGACTATAAGGATGATGACGACAAATCTAGATAGTTAATTAAACCGGTAATAAAATATCTTTATTTTCATTACATCTGTGTGTTGGTTTTTTGTGTGAATCGATAGTACTAACATA

3、USP20 (NM_006676)-human

Vector name：pCMV6-Entry

Synonyms: hVDDU2, LSFR3A, VDU2

Tag: Myc-DDK

E.coli Selection: Kanamycin

Plasmid Sequence:

TTTTGTAATACGACTCACTATAGGGCGGCCGGGAATTCGTCGACTGGATCCGGTACCGAGGAGATCTGCCGCCGCGATCGCCATGGGGGACTCCAGGGACCTTTGCCCTCACCTTGACTCCATAGGAGAGGTGACCAAAGAGGACTTGCTGCTCAAATCTAAGGGAACCTGTCAGTCGTGTGGGGTCACCGGACCAAACCTATGGGCCTGTCTGCAGGTTGCCTGCCCCTATGTTGGCTGCGGAGAATCCTTCGCTGACCACAGCACCATTCATGCACAGGCAAAAAAGCACAACTTGACCGTGAACCTGACCACGTTCCGACTGTGGTGTTACGCCTGTGAGAAGGAGGTATTCCTGGAGCAGCGGCTGGCAGCCCCTCTGCTGGGCTCCTCTTCCAAGTTCTCTGAACAGGACTCCCCGCCACCCTCCCACCCTCTGAAAGCTGTTCCTATTGCTGTGGCTGATGAAGGAGAGTCTGAGTCAGAGGATGATGACCTGAAACCTCGAGGCCTCACGGGCATGAAGAACCTCGGGAACTCCTGCTACATGAACGCCGCCCTGCAGGCCCTGTCCAATTGCCCGCCGCTGACTCAGTTCTTCTTGGAGTGTGGCGGCCTGGTGCGCACAGATAAGAAGCCAGCCCTGTGCAAGAGCTACCAGAAGCTGGTCTCTGAGGTCTGGCATAAGAAACGGCCAAGCTACGTGGTCCCCACCAGTCTGTCTCATGGGATCAAGTTGGTCAACCCAATGTTCCGAGGCTATGCCCAGCAGGACACCCAAGAGTTCCTTCGCTGCCTGATGGACCAGCTGCACGAGGAGCTCAAGGAGCCGGTGGTGGCCACGGTGGCGCTGACGGAGGCTCGGGACTCAGATTCGAGTGACACGGATGAGAAACGGGAGGGTGACCGGAGCCCATCAGAAGATGAGTTCTTGTCCTGTGACTCGAGCAGTGACCGGGGTGAGGGTGACGGGCAGGGGCGTGGCGGGGGCAGCTCGCAGGCCGAGACGGAGCTGCTGATCCCAGATGAGGCGGGCCGAGCCATCTCTGAGAAGGAGCGGATGAAGGACCGCAAGTTCTCCTGGGGCCAGCAGCGTACAAACTCGGAGCAAGTGGACGAGGACGCTGATGTGGACACTGCCATGGCTGCCCTTGACCAGCCCGCGGAGGCCCAGCCCCCGTCACCACGGTCCTCCAGCCCCTGCCGGACGCCAGAGCCGGACAATGATGCTCACCTACGCAGCTCCTCTCGCCCCTGCAGCCCCGTCCACCACCACGAGGGCCATGCCAAGCTGTCTAGCAGCCCCCCTCGTGCAAGCCCCGTGAGGATGGCACCGTCGTACGTGCTCAAGAAAGCCCAGGTATTGAGTGCTGGCAGCCGGAGGCGGAAGGAGCAGCGCTACCGCAGCGTCATCTCAGACATCTTTGACGGCTCCATTCTCAGCCTCGTGCAGTGTCTCACCTGTGACCGGGTATCCACCACAGTGGAAACGTTCCAGGACTTATCACTGCCCATTCCTGGAAAGGAGGACCTGGCCAAGCTCCATTCAGCCATCTACCAGAATGTGCCGGCCAAGCCAGGCGCCTGTGGGGACAGCTATGCCGCCCAGGGCTGGCTGGCCTTCATTGTGGAGTACATCCGACGGTTTGTGGTATCCTGTACCCCCAGCTGGTTTTGGGGGCCTGTCGTCACCCTGGAAGACTGCCTTGCTGCCTTCTTTGCCGCTGATGAGTTAAAGGGTGACAACATGTACAGCTGTGAGCGGTGTAAGAAGCTGCGGAACGGAGTGAAGTACTGCAAAGTCCTGCGGTTGCCCGAGATCCTGTGCATTCACCTAAAGCGCTTTCGGCACGAGGTGATGTACTCATTCAAGATCAACAGCCACGTCTCCTTCCCCCTCGAGGGGCTCGACCTGCGCCCCTTCCTTGCCAAGGAGTGCACATCCCAGATCACCACCTACGACCTCCTCTCGGTCATCTGCCACCACGGCACGGCAGGCAGTGGGCACTACATCGCCTACTGCCAGAACGTGATCAATGGGCAGTGGTACGAGTTTGATGACCAGTACGTCACAGAAGTCCACGAGACGGTGGTGCAGAACGCCGAGGGCTACGTACTCTTCTACAGGAAGAGCAGCGAGGAGGCCATGCGGGAGCGACAGCAGGTGGTGTCCCTGGCCGCCATGCGGGAGCCCAGCCTGCTGCGGTTCTACGTGTCCCGCGAGTGGCTCAACAAGTTCAACACCTTCGCGGAGCCAGGCCCCATCACCAACCAGACCTTCCTCTGCTCCCACGGAGGCATCCCGCCCCACAAATACCACTACATCGACGACCTGGTGGTCATCCTGCCCCAGAACGTCTGGGAGCACCTGTACAACAGATTCGGGGGTGGCCCCGCCGTGAACCACCTGTACGTGTGCTCCATCTGCCAGGTGGAGATCGAGGCACTGGCCAAGCGCAGGAGGATCGAGATCGACACCTTCATCAAGTTGAACAAGGCCTTCCAGGCCGAGGAGTCGCCGGGCGTCATCTACTGCATCAGCATGCAGTGGTTCCGGGAGTGGGAGGCGTTCGTCAAGGGGAAGGACAACGAGCCCCCCGGGCCCATTGACAACAGCAGGATTGCACAGGTCAAAGGAAGCGGCCATGTCCAGCTGAAGCAGGGAGCTGACTACGGGCAGATTTCGGAGGAGACCTGGACCTACCTGAACAGCCTGTATGGAGGTGGCCCCGAGATTGCCATCCGCCAGAGTGTGGCGCAGCCGCTGGGCCCAGAGAACCTGCACGGGGAGCAGAAGATCGAAGCCGAGACGCGGGCCGTGACGCGTACGCGGCCGCTCGAGCAGAAACTCATCTCAGAAGAGGATCTGGCAGCAAATGATATCCTGGATTACAAGGATGACGACGATAAGGTTTAA
