# Supplementary material for: LncRNA TINCR impairs the efficacy of immunotherapy against breast cancer by recruiting DNMT1 and downregulating MiR-199a-5p via the STAT1–TINCR-USP20-PD-L1 axis
Source: Cell Death Dis. 2023 Feb 1;14(2):76. doi: 10.1038/s41419-023-05609-2 (PMC9892521; doi:10.1038/s41419-023-05609-2)
Supplement: Supplementary file 6 — table S3 [file 41419_2023_5609_MOESM6_ESM.docx]

**Table. S3 The sequences of primers.**

|  | Forward Sequence | Reverse Sequence |
| --- | --- | --- |
| **Mouse** |  |  |
| TINCR | TTCCTCTCCCTTCCCTCCTT | ACATCCACTTCAGGGCTAGG |
| USP20 | CAGACAACGAAGCCCACATA | CTGAGGACAGAGCCATTGAAG |
| ACTB | ACTGCCGCATCCTCTTCCT | TCAACGTCACACTTCATGATGGA |
| **Human** |  |  |
| ACTB | CCTGTGGCATCCATGAAACTAC | CCAGGGCAGTAATCTCCTTCTG |
| TINCR | CGTGACACAAAGAGGGGAGATGACA | ACAGACTGGGGGAGCAGGAAGAG |
| PD-L1 | TGCCGACTACAAGCGAATTACTG | CTGCTTGTCCAGATGACTTCGG |
| JAK2 | ATGTCTTACCTCTTTGCTCAGTGGC | GGTTTGATCGTTTTCTTTGGCTAT |
| STAT1 | GAACTTTCTGCTGTTACTTTCCCTG | TTGGCTCTGGTGCTTCCTTTG |
| DNMT1 | AGGTGGAGAGTTATGACGAGGC | GGTAGAATGCCTGATGGTCTGC |
| USP1 | GCTCTAAAGGATGAAGCCAATCAA | ACTAGCCTGGAGCTGTTCAACC |
| USP2 | GAGATACGCACCGCGCTTTGTT | GGTTGGACTTAGGTCTCAGTGTC |
| USP12 | AGACCTTTCTGTTGACGTGGAAC | GTGTGCTTCCTGTTTGCTGCGA |
| USP19 | GCTGCTATCCTCAGAGTTGGCT | TCATCCTCCGACTGTTGCTTCC |
| USP20 | CTTTGACGGCTCCATTCTCAGC | CTCCTTTCCAGGAATGGGCAGT |
| USP33 | GTCAAAGCAGGATCATGTGGCG | CCAAAACCAGCTAGGGACACATG |
| USP35 | AGAGAACTTCCTCTCCGCATCC | CTGGACTGCTTGAGTTTCTGGC |
| USP49 | GGAGAATCTACGCTTGTGACCAG | CGGAGAACCTGAGGTAGTCTGT |
| USP54 | GAGTTAGAGGCAGCGAAAGGGT | TCTTGCAGGGACCTCTCAAAGC |
| U6 | GCTTCGGCAGCACATATACTAAAAT | CGCTTCACGAATTTGCGTGTCAT |
| miR-199a-5p | GCCCCAGTGTTCAGACTACCT | ATCCAGTGCAGGGTCCGAGG |
| pre-miR-199a-5p | TTCAGACTACCTGTTCAGGAGG | AATGTGCAGACTACTGTACACA |
| pri-miR-199a-5p | CCGCCAACCCAGTGTTCAGA | CGGGGTGGTGGAAAATGACA |
| miR-708-3p | CGGGGTGCAGCATTGTAGG | CAGCACCCGCGCACAAT |
| miR-516-5p | GGGTTCTCGAGGAAAGAA | AGTGCGTGTCGTGGAGTC |
| miR-214-3p | GCGGCACAGCAGGCACAGAC | AGCCACAAAAGAGCACAAT |
| miR-199b-3p | CGCCGACAGTAGTCTGCACA | CAGCCACAAAAGAGCACAAT |
| miR-6877-5p | GCTTCCCCAGCGGATATACTA | CGCTCGACGACCTTGCGTG |
| miR-345-3p | CGCCGGCCCTGAACGAGGGG | CAGCCACAAAAGAGCACAAT |
| let-7c-5p | CGCCGTGAGGTAGTAGGTTG | CAGCCACAAAAGAGCACAAT |
| miR-4510 | GCGGCGCAGCACCCCATG | CAGGGACAAAAGAGCAGGCA |
| miR-9-5p | CGGGCTCTTTGGTTATCTAGC | CAGCCACAAAAGAGCACAAT |
| miR-653-3p | CCAACAACAGTGTTCAGACC | CGGGGTCCTGGAAAATGACA |
| miR-15a-5p | GCGGCTAGCAGCACATAAT | CAGCCACAAAAGAGCACAAT |
| miR-195-5p | GCGGCTAGCAGCACAGAAA | CAGCCACAAAAGAGCACAAT |
| miR-107 | CGGGCAGCAGCATTGTACAGG | CAGCCACAAAAGAGCACAAT |
| miR-424-5p | CAGCAGCAAUUCAUGUUUUGAA | CAGCCACAAAAGAGCACAAT |
| miR-16-5p | GCGGCTAGCAGCACGTAAAT | CAGCCACAAAAGAGCACAAT |
| miR-627-3p | CACTCATCTTTTCTTTG | GAGTCTCTTGAGAGT ACAT |
| miR-149-5p | CGCCGTCTGGCTCCGTGTCTT | CAGCCACAAAAGAGCACAAT |
| miR-103a-3p | CGCCGAGCAGCATTGTACAGG | CAGCCACAAAAGAGCACAAT |
| miR-15b-5p | GCGGCTAGCAGCACATCATG | CAGCCACAAAAGAGCACAAT |
| miR-125b-5p | CGCCGTCCCTGAGACCCTAA | CAGCCACAAAAGAGCACAAT |
| let-7e-5p | CATTCTCTCAGAGGAGGTAGGAGG | TATGGTTTTGACGACTGTGTGAT |
| miR-378 | GCACTGGACTTGGAGTC | GTGCAGGGTCGAGGT |
| miR-30b-3p | AGGTGTTCAGCTGAGTGTAGG | CAGTGCAGGGTCCGAGGT |
| miR-125a-5p | GCTCCCTGAGACCCTTTA | CAGTGCGTGTCGTGGAGT |
