# Supplementary material for: LncRNA TINCR impairs the efficacy of immunotherapy against breast cancer by recruiting DNMT1 and downregulating MiR-199a-5p via the STAT1–TINCR-USP20-PD-L1 axis
Source: Cell Death Dis. 2023 Feb 1;14(2):76. doi: 10.1038/s41419-023-05609-2 (PMC9892521; doi:10.1038/s41419-023-05609-2)
Supplement: Supplementary file 8 — table S5 [file 41419_2023_5609_MOESM8_ESM.docx]

**Table. S5 There senses of TINCR used in the RNA pulldown.**

**TINCR sense1**

GggcgggcggagcgcgggcgcggcgggggcgggcggccaggctagtcgggcgggtgcgcggggcgctcggggcccggggccagagctggagccggagccgggcgggcgccatggaggggctgcggcgggggctgtcgcgctggaagcgctaccacatcaaggtgcacctggcggacgaggcgctgctgctaccgctgaccgtgcggccgcgggacacgctcagcgacctgcgcgcccagctggtgggccagggcgtgagctcctggaagcgcgccttctactacaacgcgcggcggctggacgaccaccagacggtgcgcgacgcgcgcctgcaggacggctcggtgctgctgctcgtcagcgaccccaggtagtctgggttggaggaggcagagccatgaccaaggggacctgggtactggctgaaggaataggctggggtagagggcacttttggaaggcacttctcctgcctcccgggagcctagatctcactccagggtctgggctcccaggtggaccatgaaaccctggcctgaccagctgatgcacactgcttcagacactcctgctggagccccagtccctgacaaggacctaggacatttttgctcctgcccagcctatcgggagggagccttgagcctttcagctctgctgtgtgactttgaggttgttgctcccctcttggggccctgggtgccctgtcttcagtggaaagcactgtgccaccttggaaagctcccatgggcagccagagggcatcgcaagaagagaagcacagaaggggcaggagagacactcagaggcacttccgctcttgcccaggacattttcccagccacacctttgcccaagccgtgccccctgcctggagcacttttcaacctcttctctgcagctccaatacacctgggattgcagtctcctccaggaagtcttctcagattccctccttcccagccagagagcacctagccttctttggggcccccacaggccctttgtgcagtgaacagccctggctgggggtgcagccagtcgtgtccgaactctccaatgactaagcggggagatgcggacatcctagctccttctcaggcctccaactgtgccccatttccacccccaaatacctccccaggaggcacctgtgcccaccccctgggctgtttcccccttccccttaaatccggatgcctcgtcttgcatagggtcttggggcagcggggaaGgggttctgaagaact

**TINCR sense2**

Ggggttctgaagaactctggccaagaggacgaggatctgggggtggggaactgggcctagctgtgggaggtcatcgcggggcattgcagggagtgcgttgtgggaattccgggtggagaccctcagggcagtgtgccgggcctccgttgcacctctgacctgcagccacgtcatcgttgcgcagcccctggggagggtctttggggagggggtgccgaggctacgcatgtcctcgagggccgtgtacactctccaggcaccatgggcggaggcgccagaggctgggaagaaataatgttttagttaagagtcctgttggctgcaggactcagagcatggacaggtggatagtaaatcaccaccacggggacagccgtgcccagactgtgcgtttgcttagctcggggacagcacttggcccggggtctcctgctcgcctcccttcagagcatctgccaaacttcgggcatctaccctgcaattcccgcttggctgagaggagggggagaaggaggggaagagagaggagccccacttcactccggcaggcagacctgctggagctgctttgcagaatgacttgggtcttgctggcccctgggtgtgcctggaggggggtcttcctatccccctccacctctccccttggtgcctaacccaggactttgtccccagagacccactgtgtgccccctggagctcctcctagagcagagtctgctgctgtctgttctgcagagggtagcctgaggcccaaggaggttgtcagggacacacagcagggggaggcagcccagatccctgctcatgcgtggtttgggcctctgctaacagctgggccacatgcgtgtatctagagcctgggtcccctctgtcctcatttccctgtgcatattcagggacatgcctcgtcagggtttggcaagaaaatggggcatttaataatctccccagtttcattccttggtccttactccatgccaggacttgtgcacatctttttggagccctgtctcacttgattgacagccggccaggcgcggtggctcacgcctgtaatcccagcactttgggaggacgaggcaggcggatcatgaggtcaggagatggagaccatcctggctaacacggtgaaaccccgtctctactaaaagtacaaaaaaaatagccgggcctggtggcgggcgcctgtagtcccagctacttggtaggctgaggcaggagaatggcgggaacccgggaggcagaggttgcagtgagctgag

**TINCR sense3**

gttgcagtgagctgagatcacgccactgcactccagcctgggcgacagagcgagactccgtctcaaaaaaaaaaaaaaaaaagattgacagcctcactgctcaggggccaggataacagcccattttacagatggcttttggcttgcccaaggtcaccaccctctgaactgaggcgtccccaacccatgccgggagctggtggaggaagtggggaagggtttcccgcttctccactgggggtggaggtgggagaaagcattttcagggtgttttgacatttaacaaacagtatgcaaatcacatgcaaatcgcatgcaaatcaccacccatttcttaagcctcgacagcgctggcatgttctgaaataataatgttggacctgtcccttgatgatgacctgggtcgggaggtgcacggccttttccttcctccgtcttcccttcccttcccttcccttcccatctgttctcccttccttcgcccccttcctttcctctccctccctatcttccatttttctcttcttccttcacatcatttgtccaagacctccctggcctgagataaacataagttgcaaaagtggcttcctgcagcccattttccaggttgggaaactgaggcatgggcctaaggtcccacagccacaccgaggaaagcagcccccactgaatgtcaccatgcaccaaagtgcatggtcctcgtgacacaaagaggggagatgacagtggctggagttgtcagagctgctttgaggaagctccaggcctgataggcttgacagggccaaggggaactattgtggaatgtcttggccttgaatgacaggctgctgattttggatctggagaaagctcacactgactcttcctgctcccccagtctgtttttctcctgctactccatccatgtatccctgagtttgggccacagtccctccacttggtttgcatgtcccaccttctttgtttggcaagctcctattcatccttcaaagccctagcttcaatacctgctacttcatgcagccattgcttatccttcaaggagagcctacttccctcaaggaccctgctgcttccagtctcaccttctgactcactcgggatccactgaactgggaggtctgtgtctcctcccagcagagtcatcactacctttggggccgcaggatcacccagcttggaactagatacagaaatgctgttttgagagtgtactgaataaaagattacatgtttgaaaacaa
