# Supplementary material for: LncRNA TINCR impairs the efficacy of immunotherapy against breast cancer by recruiting DNMT1 and downregulating MiR-199a-5p via the STAT1–TINCR-USP20-PD-L1 axis
Source: Cell Death Dis. 2023 Feb 1;14(2):76. doi: 10.1038/s41419-023-05609-2 (PMC9892521; doi:10.1038/s41419-023-05609-2)
Supplement: Supplementary file 9 — table S6 [file 41419_2023_5609_MOESM9_ESM.doc]

**Table. S6 The combination sequence and primers of TINCR and STAT1.**

**Target1**

**Sequence：tttctctctgtttcctctgggaaa**

**Primer：**STAT1-TINCR T1F: CTAGGTGGCCTGGGTTCGA

STAT1-TINCR T1R: AGAAAACTGAGGCATAGAGAAGAAAAG

**Target2**

**Sequence：gctcttttcttttcttttct**

**Primer：**STAT1-TINCR T2F: TCTGTCACTTAGCAGGTGCTCTTT

STAT1-TINCR T2R: GTGAGCGGAGATCTGCCATT
