# Supplementary material for: LncRNA TINCR impairs the efficacy of immunotherapy against breast cancer by recruiting DNMT1 and downregulating MiR-199a-5p via the STAT1–TINCR-USP20-PD-L1 axis
Source: Cell Death Dis. 2023 Feb 1;14(2):76. doi: 10.1038/s41419-023-05609-2 (PMC9892521; doi:10.1038/s41419-023-05609-2)
Supplement: Supplementary file 10 — table S7 [file 41419_2023_5609_MOESM10_ESM.docx]

**Table. S7 The mutation sequences of TINCR promoter.**

TINCR-promoter

TGAGGCAGGAGAATTGCTTGCACCTGGGAGGCGGAGGTTGCGGTGAGCCGAGATTGTGCCATTGTACTCCACCCTGGGCAACAAGAGTGAAACTCTGTCTCAAAAAAAAAAAAAAAAAAAAAAGTGATGTTTGTGTAATGAATGGATTACAACAAAACTGCTAATGCTAGGTGGCCTGGGTTCGAATCCCAGCTCTGCCACTTTTCTCTCTGTTTCCTCTGGGAAATCTCTTTTCTTCTCTATGCCTCAGTTTTCTCACTTGTAAAATGGGAAGTCATAGTGTCATGAGAAAAGTAAGAGATGCTTCCCCCACCCCAATAAAGTACCTAGAAGACTGTCTGTCACTTAGCAGGTGCTCTTTTCTTTTCTTTTCTTTTTTGAGACGGAGTTTCCCTCTTGTTGCCCAGGCTAGAGTGCAATGGCAGATCTCCGCTCACCACAACCTCCACCTCCTGGGTTCAAGCGATTCTCCTGGCTCAGCCTCCTGAGTAGTTGGGATTACAGGCACCCACCACCACGCCTGGCTAATTTTTTTGTATTTTTTGTAGAGACGGGGTTTCTCTGTGTTGGTCAGGTAGATCTCGATCTCCCAACCTCAGGTGATCTGCCCACCTCGGCCTCCCAAAGTGTTGGGATTACAGGCGTTAGCCCCTGCACCCAGCCCTTCTCTTTTTTTCCTTCTCTTCCTCTCTCTCTCTCTTTCTTTCTTTCATTCTTTCTTTCCTCTTCTCTTTCTCTCTCTCTCCCTCTTTCTTTCTTTCTTTCTTTCTTTTTTTTTTTTTTTTTTGAGACGGAGTCTCGCTGTGTCTCCCAGGCTGGAATGCAGTGGCGCACGATCTCGGCTCACTGCAAGCTCCGCCTCCCAGGTTCACGCCATTCTCTTGCCTCAGCCTCCGGAGTAGCTGGGACTACAGGCGCCCGCCACCACGCCCGGCTAATTTTTTGTATTTTTGGTAGAGACGGGGGGGTTTCACCGCGTTAGCCAGGATGGTCTCGATCTCCTGACCTTGTGATCTGCCCACCTGGGCCTCCCAAAGTACTGGGATTACAGGCGTGAGCCACCACACCCGGCCATTTTTTTTTTTTTTTTTTTTTTTTTTTTTTTTGCGATAAGTCTCGCTCTGTGGCTCAGGCTGGAGTGCAGTGGCACAATCTCGGCTCACTGCAACCTCTGTCTGCGGGTTCAAGCGATTCTTCTGCCTCAGCCTCCTGAGTAGCTGGAACTACAGGCATGTGCCACCACGCCTGGCTAATTTTTGTACTTATAGTAGAGACGGGGTTTCACCATGTTGGCCAGGTTGGTCTCGAACTCCTGACCTCATGTGATCCGCCCACCTCAGTCTCCCAAAGTGCTGGGATTCCAGGCATGAGCCACCGCACCTGGCCTAAAAGTAAATTTTTTTTTTAATGTAGGCTGAGCGTGGTGGCTGAGGGTTATAATCCCACCGCTTTGGGAGGCCAAGGCGGAGAATCACTTGACGTCAGGAGTTCAAAACCAGCCTGGGCAACACGGCCAGGCCCCATCTCTACAAAAACTACAAAAGTGAGCTGGGCTTGGTGGCAGCGCCTGTAGTCCCAGCTACTTGAGAGGCTGAGGCGGGAGGATCGCTTGAGCCCGGGAGGTCAAGGCTGCAACGAACCATGATTGCGCCACTGCGCTCCAGCCTGTCCTTAAAAAGATCAAATAAAGTGGCCTCCCAGCCTCCCTCCCTGGAGCCTCCACCATGTAACCCCCTGGACATTCGCGCGTCTCCTTGGCTGGGATGACCTCGCTGATGGCTCTGTGCTGAGTGGCCACTCCGCCTCTAGACCCCCGGGCGCACTCTGGGGCCAGCAGTGGGGAGCGGACGCCTGAGGGGACCGTGGCACCCGCCCCGCCCCGCCGGGCCGGGCCTGCCCACTGCGTGCCGGGCGTGTCCGGGCGGGCGGAGCGCGGGCGCGGCGGGGGCGGGCGGCCAGGCTAGTCGGGCGGGTGCGCGGGGCGCTCGGGGCCCGGGGCCAGAGCTGGAGCCGGAGCCGGGCGGGCGCC

TINCR-promoter-MUT1

TGAGGCAGGAGAATTGCTTGCACCTGGGAGGCGGAGGTTGCGGTGAGCCGAGATTGTGCCATTGTACTCCACCCTGGGCAACAAGAGTGAAACTCTGTCTCAAAAAAAAAAAAAAAAAAAAAAGTGATGTTTGTGTAATGAATGGATTACAACAAAACTGCTAATGCTAGGTGGCCTGGGTTCGAATCCCAGCTCTGCCACTTGGCGAAAACCCGGGGGGGGGGGGGGTCTTTTCTTCTCTATGCCTCAGTTTTCTCACTTGTAAAATGGGAAGTCATAGTGTCATGAGAAAAGTAAGAGATGCTTCCCCCACCCCAATAAAGTACCTAGAAGACTGTCTGTCACTTAGCAGGTGCTCTTTTCTTTTCTTTTCTTTTTTGAGACGGAGTTTCCCTCTTGTTGCCCAGGCTAGAGTGCAATGGCAGATCTCCGCTCACCACAACCTCCACCTCCTGGGTTCAAGCGATTCTCCTGGCTCAGCCTCCTGAGTAGTTGGGATTACAGGCACCCACCACCACGCCTGGCTAATTTTTTTGTATTTTTTGTAGAGACGGGGTTTCTCTGTGTTGGTCAGGTAGATCTCGATCTCCCAACCTCAGGTGATCTGCCCACCTCGGCCTCCCAAAGTGTTGGGATTACAGGCGTTAGCCCCTGCACCCAGCCCTTCTCTTTTTTTCCTTCTCTTCCTCTCTCTCTCTCTTTCTTTCTTTCATTCTTTCTTTCCTCTTCTCTTTCTCTCTCTCTCCCTCTTTCTTTCTTTCTTTCTTTCTTTTTTTTTTTTTTTTTTGAGACGGAGTCTCGCTGTGTCTCCCAGGCTGGAATGCAGTGGCGCACGATCTCGGCTCACTGCAAGCTCCGCCTCCCAGGTTCACGCCATTCTCTTGCCTCAGCCTCCGGAGTAGCTGGGACTACAGGCGCCCGCCACCACGCCCGGCTAATTTTTTGTATTTTTGGTAGAGACGGGGGGGTTTCACCGCGTTAGCCAGGATGGTCTCGATCTCCTGACCTTGTGATCTGCCCACCTGGGCCTCCCAAAGTACTGGGATTACAGGCGTGAGCCACCACACCCGGCCATTTTTTTTTTTTTTTTTTTTTTTTTTTTTTTTGCGATAAGTCTCGCTCTGTGGCTCAGGCTGGAGTGCAGTGGCACAATCTCGGCTCACTGCAACCTCTGTCTGCGGGTTCAAGCGATTCTTCTGCCTCAGCCTCCTGAGTAGCTGGAACTACAGGCATGTGCCACCACGCCTGGCTAATTTTTGTACTTATAGTAGAGACGGGGTTTCACCATGTTGGCCAGGTTGGTCTCGAACTCCTGACCTCATGTGATCCGCCCACCTCAGTCTCCCAAAGTGCTGGGATTCCAGGCATGAGCCACCGCACCTGGCCTAAAAGTAAATTTTTTTTTTAATGTAGGCTGAGCGTGGTGGCTGAGGGTTATAATCCCACCGCTTTGGGAGGCCAAGGCGGAGAATCACTTGACGTCAGGAGTTCAAAACCAGCCTGGGCAACACGGCCAGGCCCCATCTCTACAAAAACTACAAAAGTGAGCTGGGCTTGGTGGCAGCGCCTGTAGTCCCAGCTACTTGAGAGGCTGAGGCGGGAGGATCGCTTGAGCCCGGGAGGTCAAGGCTGCAACGAACCATGATTGCGCCACTGCGCTCCAGCCTGTCCTTAAAAAGATCAAATAAAGTGGCCTCCCAGCCTCCCTCCCTGGAGCCTCCACCATGTAACCCCCTGGACATTCGCGCGTCTCCTTGGCTGGGATGACCTCGCTGATGGCTCTGTGCTGAGTGGCCACTCCGCCTCTAGACCCCCGGGCGCACTCTGGGGCCAGCAGTGGGGAGCGGACGCCTGAGGGGACCGTGGCACCCGCCCCGCCCCGCCGGGCCGGGCCTGCCCACTGCGTGCCGGGCGTGTCCGGGCGGGCGGAGCGCGGGCGCGGCGGGGGCGGGCGGCCAGGCTAGTCGGGCGGGTGCGCGGGGCGCTCGGGGCCCGGGGCCAGAGCTGGAGCCGGAGCCGGGCGGGCGCC

TINCR-promoter-MUT2

TGAGGCAGGAGAATTGCTTGCACCTGGGAGGCGGAGGTTGCGGTGAGCCGAGATTGTGCCATTGTACTCCACCCTGGGCAACAAGAGTGAAACTCTGTCTCAAAAAAAAAAAAAAAAAAAAAAGTGATGTTTGTGTAATGAATGGATTACAACAAAACTGCTAATGCTAGGTGGCCTGGGTTCGAATCCCAGCTCTGCCACTTTTCTCTCTGTTTCCTCTGGGAAATCTCTTTTCTTCTCTATGCCTCAGTTTTCTCACTTGTAAAATGGGAAGTCATAGTGTCATGAGAAAAGTAAGAGATGCTTCCCCCACCCCAATAAAGTACCTAGAAGACTGTCTGTCACTTAGACGGGAACCCAGTTGGGAACCCAGTTTTTTGAGACGGAGTTTCCCTCTTGTTGCCCAGGCTAGAGTGCAATGGCAGATCTCCGCTCACCACAACCTCCACCTCCTGGGTTCAAGCGATTCTCCTGGCTCAGCCTCCTGAGTAGTTGGGATTACAGGCACCCACCACCACGCCTGGCTAATTTTTTTGTATTTTTTGTAGAGACGGGGTTTCTCTGTGTTGGTCAGGTAGATCTCGATCTCCCAACCTCAGGTGATCTGCCCACCTCGGCCTCCCAAAGTGTTGGGATTACAGGCGTTAGCCCCTGCACCCAGCCCTTCTCTTTTTTTCCTTCTCTTCCTCTCTCTCTCTCTTTCTTTCTTTCATTCTTTCTTTCCTCTTCTCTTTCTCTCTCTCTCCCTCTTTCTTTCTTTCTTTCTTTCTTTTTTTTTTTTTTTTTTGAGACGGAGTCTCGCTGTGTCTCCCAGGCTGGAATGCAGTGGCGCACGATCTCGGCTCACTGCAAGCTCCGCCTCCCAGGTTCACGCCATTCTCTTGCCTCAGCCTCCGGAGTAGCTGGGACTACAGGCGCCCGCCACCACGCCCGGCTAATTTTTTGTATTTTTGGTAGAGACGGGGGGGTTTCACCGCGTTAGCCAGGATGGTCTCGATCTCCTGACCTTGTGATCTGCCCACCTGGGCCTCCCAAAGTACTGGGATTACAGGCGTGAGCCACCACACCCGGCCATTTTTTTTTTTTTTTTTTTTTTTTTTTTTTTTGCGATAAGTCTCGCTCTGTGGCTCAGGCTGGAGTGCAGTGGCACAATCTCGGCTCACTGCAACCTCTGTCTGCGGGTTCAAGCGATTCTTCTGCCTCAGCCTCCTGAGTAGCTGGAACTACAGGCATGTGCCACCACGCCTGGCTAATTTTTGTACTTATAGTAGAGACGGGGTTTCACCATGTTGGCCAGGTTGGTCTCGAACTCCTGACCTCATGTGATCCGCCCACCTCAGTCTCCCAAAGTGCTGGGATTCCAGGCATGAGCCACCGCACCTGGCCTAAAAGTAAATTTTTTTTTTAATGTAGGCTGAGCGTGGTGGCTGAGGGTTATAATCCCACCGCTTTGGGAGGCCAAGGCGGAGAATCACTTGACGTCAGGAGTTCAAAACCAGCCTGGGCAACACGGCCAGGCCCCATCTCTACAAAAACTACAAAAGTGAGCTGGGCTTGGTGGCAGCGCCTGTAGTCCCAGCTACTTGAGAGGCTGAGGCGGGAGGATCGCTTGAGCCCGGGAGGTCAAGGCTGCAACGAACCATGATTGCGCCACTGCGCTCCAGCCTGTCCTTAAAAAGATCAAATAAAGTGGCCTCCCAGCCTCCCTCCCTGGAGCCTCCACCATGTAACCCCCTGGACATTCGCGCGTCTCCTTGGCTGGGATGACCTCGCTGATGGCTCTGTGCTGAGTGGCCACTCCGCCTCTAGACCCCCGGGCGCACTCTGGGGCCAGCAGTGGGGAGCGGACGCCTGAGGGGACCGTGGCACCCGCCCCGCCCCGCCGGGCCGGGCCTGCCCACTGCGTGCCGGGCGTGTCCGGGCGGGCGGAGCGCGGGCGCGGCGGGGGCGGGCGGCCAGGCTAGTCGGGCGGGTGCGCGGGGCGCTCGGGGCCCGGGGCCAGAGCTGGAGCCGGAGCCGGGCGGGCGCC

TINCR-promoter-MUT

TGAGGCAGGAGAATTGCTTGCACCTGGGAGGCGGAGGTTGCGGTGAGCCGAGATTGTGCCATTGTACTCCACCCTGGGCAACAAGAGTGAAACTCTGTCTCAAAAAAAAAAAAAAAAAAAAAAGTGATGTTTGTGTAATGAATGGATTACAACAAAACTGCTAATGCTAGGTGGCCTGGGTTCGAATCCCAGCTCTGCCACTTGGCGAAAACCCGGGGGGGGGGGGGGTCTTTTCTTCTCTATGCCTCAGTTTTCTCACTTGTAAAATGGGAAGTCATAGTGTCATGAGAAAAGTAAGAGATGCTTCCCCCACCCCAATAAAGTACCTAGAAGACTGTCTGTCACTTAGACGGGAACCCAGTTGGGAACCCAGTTTTTTGAGACGGAGTTTCCCTCTTGTTGCCCAGGCTAGAGTGCAATGGCAGATCTCCGCTCACCACAACCTCCACCTCCTGGGTTCAAGCGATTCTCCTGGCTCAGCCTCCTGAGTAGTTGGGATTACAGGCACCCACCACCACGCCTGGCTAATTTTTTTGTATTTTTTGTAGAGACGGGGTTTCTCTGTGTTGGTCAGGTAGATCTCGATCTCCCAACCTCAGGTGATCTGCCCACCTCGGCCTCCCAAAGTGTTGGGATTACAGGCGTTAGCCCCTGCACCCAGCCCTTCTCTTTTTTTCCTTCTCTTCCTCTCTCTCTCTCTTTCTTTCTTTCATTCTTTCTTTCCTCTTCTCTTTCTCTCTCTCTCCCTCTTTCTTTCTTTCTTTCTTTCTTTTTTTTTTTTTTTTTTGAGACGGAGTCTCGCTGTGTCTCCCAGGCTGGAATGCAGTGGCGCACGATCTCGGCTCACTGCAAGCTCCGCCTCCCAGGTTCACGCCATTCTCTTGCCTCAGCCTCCGGAGTAGCTGGGACTACAGGCGCCCGCCACCACGCCCGGCTAATTTTTTGTATTTTTGGTAGAGACGGGGGGGTTTCACCGCGTTAGCCAGGATGGTCTCGATCTCCTGACCTTGTGATCTGCCCACCTGGGCCTCCCAAAGTACTGGGATTACAGGCGTGAGCCACCACACCCGGCCATTTTTTTTTTTTTTTTTTTTTTTTTTTTTTTTGCGATAAGTCTCGCTCTGTGGCTCAGGCTGGAGTGCAGTGGCACAATCTCGGCTCACTGCAACCTCTGTCTGCGGGTTCAAGCGATTCTTCTGCCTCAGCCTCCTGAGTAGCTGGAACTACAGGCATGTGCCACCACGCCTGGCTAATTTTTGTACTTATAGTAGAGACGGGGTTTCACCATGTTGGCCAGGTTGGTCTCGAACTCCTGACCTCATGTGATCCGCCCACCTCAGTCTCCCAAAGTGCTGGGATTCCAGGCATGAGCCACCGCACCTGGCCTAAAAGTAAATTTTTTTTTTAATGTAGGCTGAGCGTGGTGGCTGAGGGTTATAATCCCACCGCTTTGGGAGGCCAAGGCGGAGAATCACTTGACGTCAGGAGTTCAAAACCAGCCTGGGCAACACGGCCAGGCCCCATCTCTACAAAAACTACAAAAGTGAGCTGGGCTTGGTGGCAGCGCCTGTAGTCCCAGCTACTTGAGAGGCTGAGGCGGGAGGATCGCTTGAGCCCGGGAGGTCAAGGCTGCAACGAACCATGATTGCGCCACTGCGCTCCAGCCTGTCCTTAAAAAGATCAAATAAAGTGGCCTCCCAGCCTCCCTCCCTGGAGCCTCCACCATGTAACCCCCTGGACATTCGCGCGTCTCCTTGGCTGGGATGACCTCGCTGATGGCTCTGTGCTGAGTGGCCACTCCGCCTCTAGACCCCCGGGCGCACTCTGGGGCCAGCAGTGGGGAGCGGACGCCTGAGGGGACCGTGGCACCCGCCCCGCCCCGCCGGGCCGGGCCTGCCCACTGCGTGCCGGGCGTGTCCGGGCGGGCGGAGCGCGGGCGCGGCGGGGGCGGGCGGCCAGGCTAGTCGGGCGGGTGCGCGGGGCGCTCGGGGCCCGGGGCCAGAGCTGGAGCCGGAGCCGGGCGGGCGCC
