# Supplementary material for: LncRNA TINCR impairs the efficacy of immunotherapy against breast cancer by recruiting DNMT1 and downregulating MiR-199a-5p via the STAT1–TINCR-USP20-PD-L1 axis
Source: Cell Death Dis. 2023 Feb 1;14(2):76. doi: 10.1038/s41419-023-05609-2 (PMC9892521; doi:10.1038/s41419-023-05609-2)
Supplement: Supplementary file 12 — Supplementary figure and table legends [file 41419_2023_5609_MOESM12_ESM.docx]

**Supplementary Figure Legends**

**Fig. S1 Knockdown efficiency of TINCR.** A. TINCR expression in 4T1 cells. B. TINCR expression in MDA-MB-231 cells. C. TINCR expression in UACC812 cells. Data are presented as means from three independent experiments ± S.D. **P* < 0.05, ****P* < 0.001, *****P* < 0.0001.

**Fig. S2 TINCR upregulates PD-L1 by inhibiting its degradation.** T47D cells were treated with MG132 (10 µM) to detect PD-L1 expression in the control and TINCR knockdown groups.

**Fig. S3 TINCR upregulates the expression of USP20 in vivo and in vitro. A-B** mRNA expression of USP20 after TINCR knockdown in MDA-MB-231 and UACC812 cells. **C, D** The mRNA expression of USP20 after TINCR knockdown in mouse tissue. Data are presented as means from three independent experiments ± S.D. **P* < 0.05, ***P* < 0.01, ****P* < 0.001.

**Fig. S4 Enrichment peak of DNMT1 in the promoter of miR-199a.** Using the ENCODE database to predict the binding position of DNMT1 on miR-199a.

**Fig. S5 TINCR could dock to DNMT1.** We used the Hex 8.0 to predict the docking result between TINCR and DNMT1.

**Fig. S6 TINCR could interact with DNMT1.** Using the RNA-pulldown, three sequences of TINCR could pull down DNMT1.

**Fig. S7 The expression of TINCR and DNMT1.** In the rescue assay, the expression of TINCR and DNMT1 was detected by qPCR. Data are presented as means from three independent experiments ± S.D. ***P* < 0.01, ****P* < 0.001.

**Fig. S8 IFN-γ promotes the progression of breast cancer in vitro. A-C** IFN-γ promotes (**A**) migration, (**B**) invasiveness and (**C**) proliferation in breast cancer cells. Data are presented as means from three independent experiments ± S.D. **P* < 0.05, ***P* < 0.01, ****P* < 0.001, *****P* < 0.0001.

**Fig. S9 The expression of miR-199a-5p after IFN-γ stimulation.** Data are presented as means from three independent experiments ± S.D. ***P* < 0.01, ***P* < 0.01, *****P* < 0.0001.

**Fig. S10 Enrichment peak of STAT1 in the promoter of TINCR. A** Upper corner of picture: STAT1-binding motif; lower table: prediction of STAT1-binding sites within TINCR promoter region, from the JASPAR database. **B** Enrichment peak of STAT1 in the promoter of TINCR using ChIP-seq data from the ENCODE database.

**Supplementary Table Legends**

**Table. S1 The sequences of siRNA and miR-199a-5p mimic or inhibitor.**

**Table. S2 The information of plasmids.**

**Table. S3 The sequences of primers.**

**Table. S4 The clinical characteristics of the breast cancer samples in Fig 2A.**

**Table. S5 There senses of TINCR used in the RNA pulldown.**

**Table. S6 The combination sequence and primers of TINCR and STAT1.**

**Table. S7 The mutation sequences of TINCR promoter.**

**Table. S8 The clinical characteristics of the breast cancer samples in Fig 6O.**
