# Supplementary material for: LncRNA TINCR impairs the efficacy of immunotherapy against breast cancer by recruiting DNMT1 and downregulating MiR-199a-5p via the STAT1–TINCR-USP20-PD-L1 axis
Source: Cell Death Dis. 2023 Feb 1;14(2):76. doi: 10.1038/s41419-023-05609-2 (PMC9892521; doi:10.1038/s41419-023-05609-2)

Figure 2C

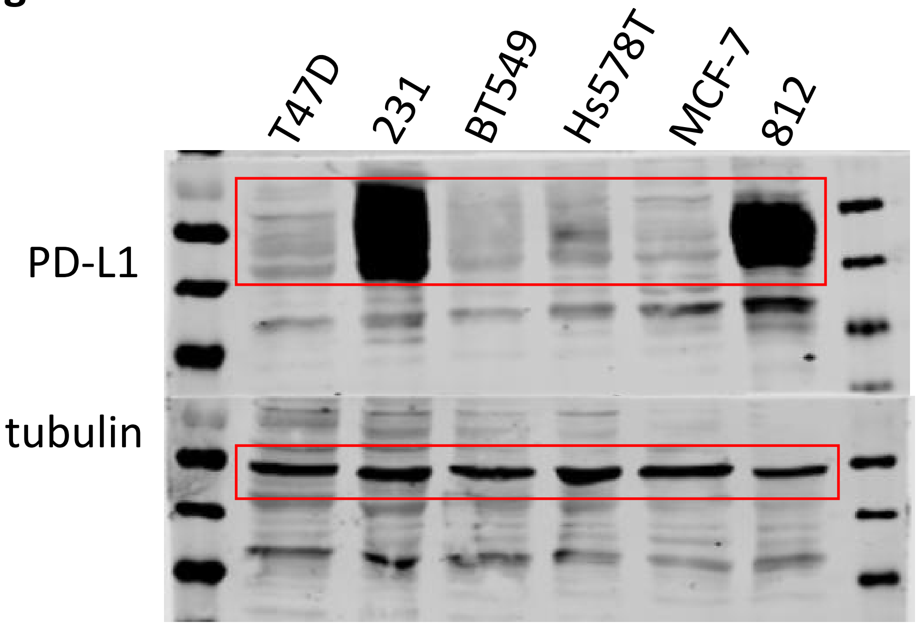

Figure 2D

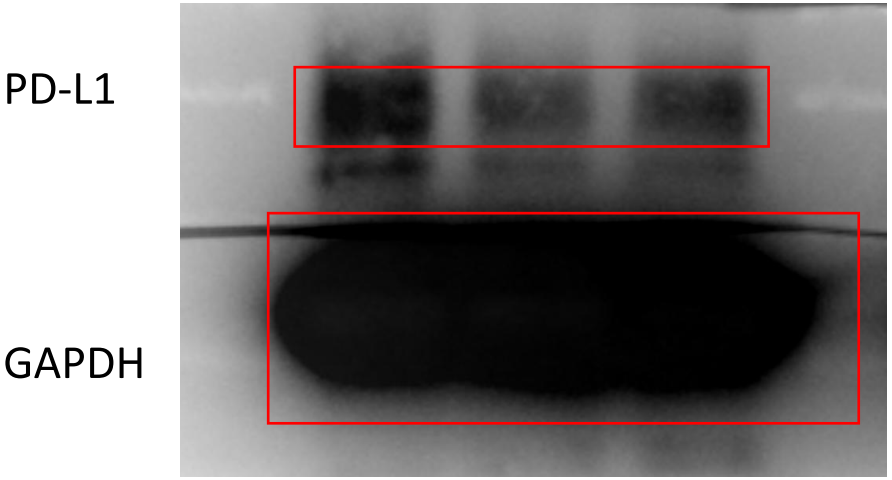

Figure 2E

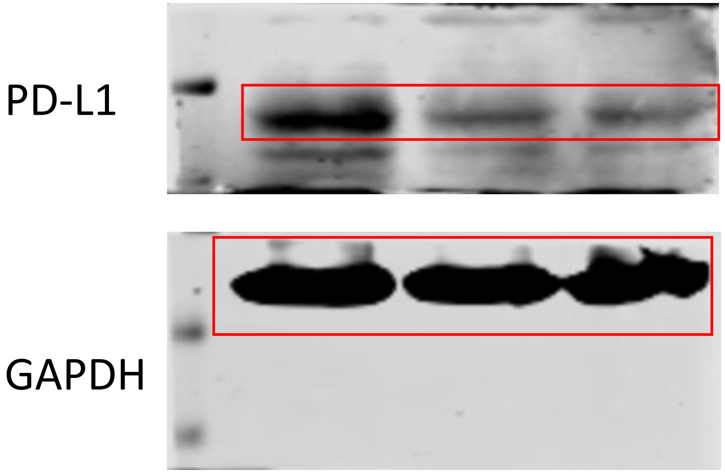

Figure 2F

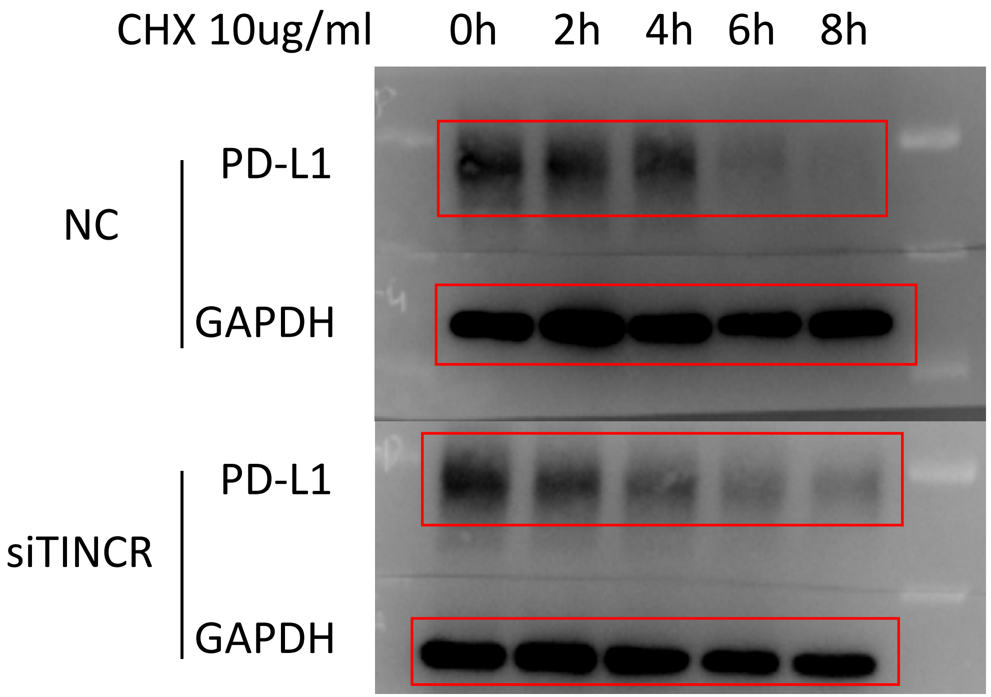

Figure 2G

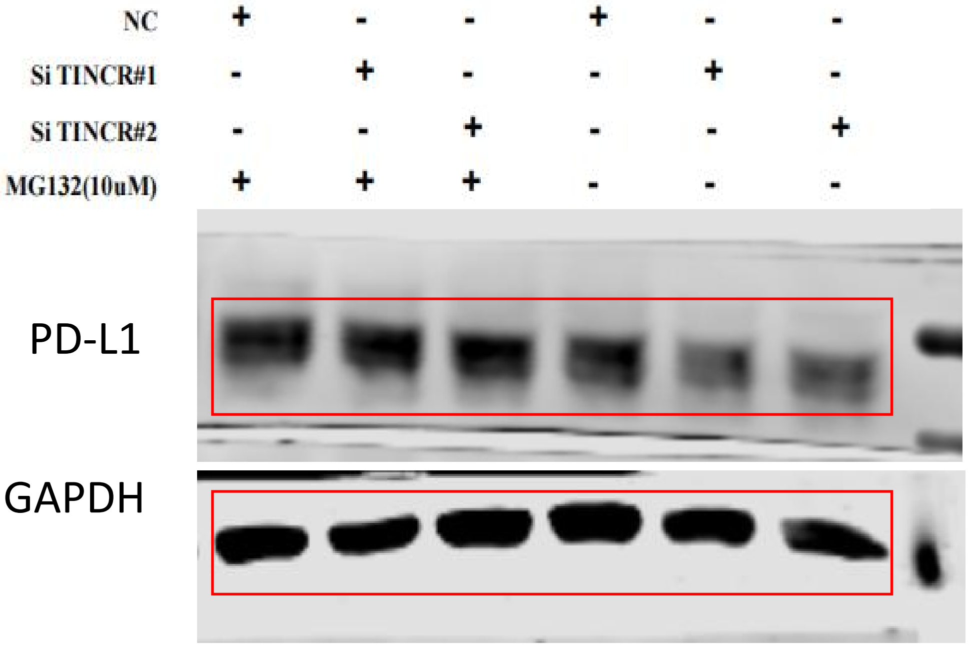

Figure 2H

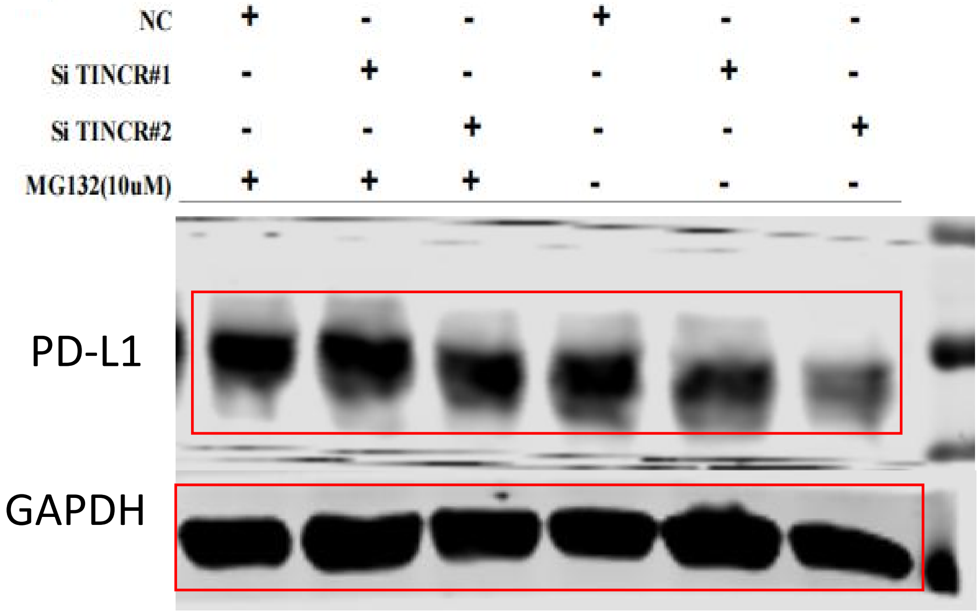

Figure 3D

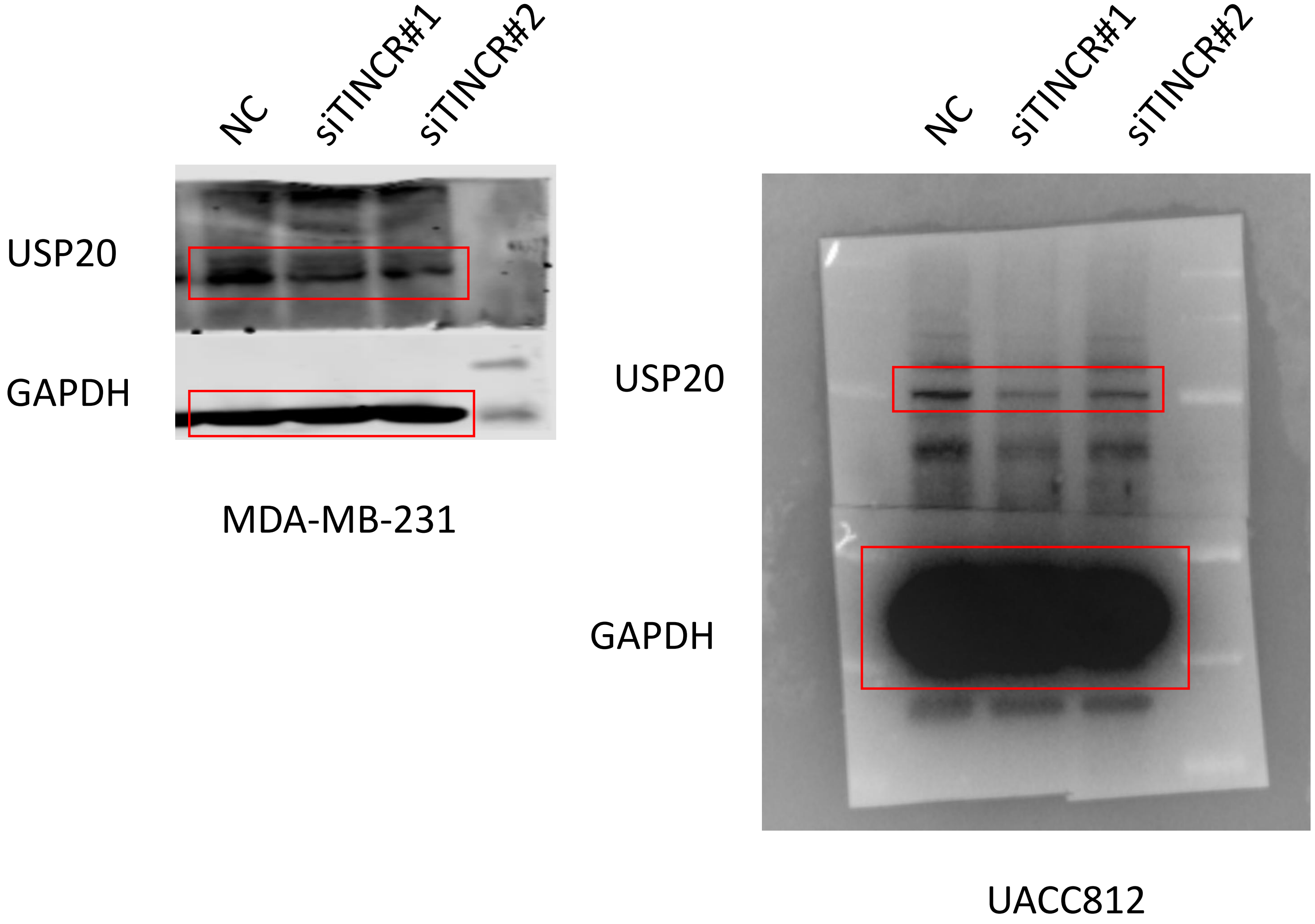
NC
siTINCR#1
siTINCR#2
USP20
GAPDH
MDA-MB-231
NC
siTINCR#1
siTINCR#2
USP20
GAPDH
UACC812

Figure 3E

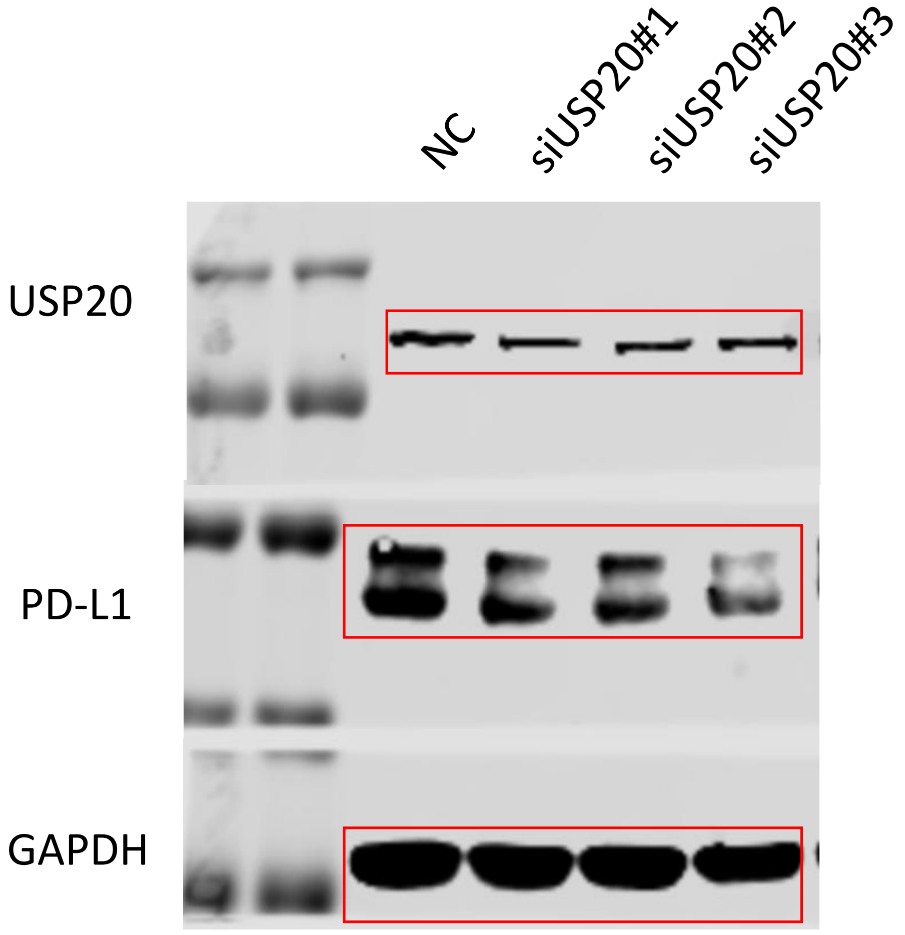

Figure 3F

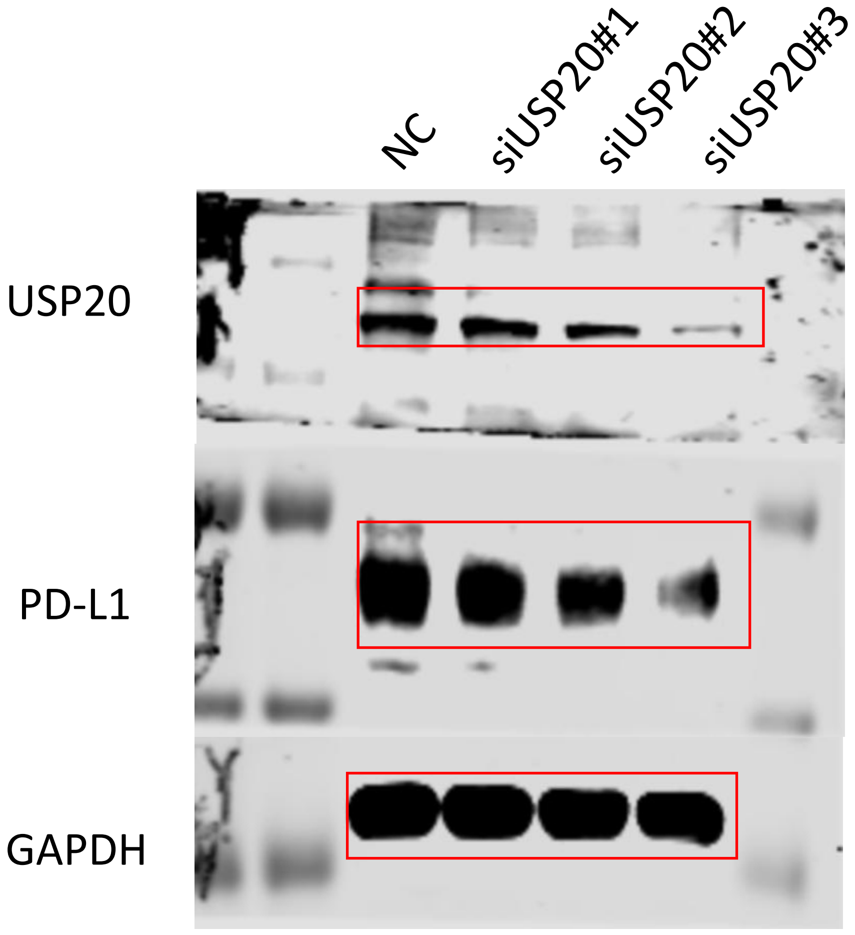

Figure 3H

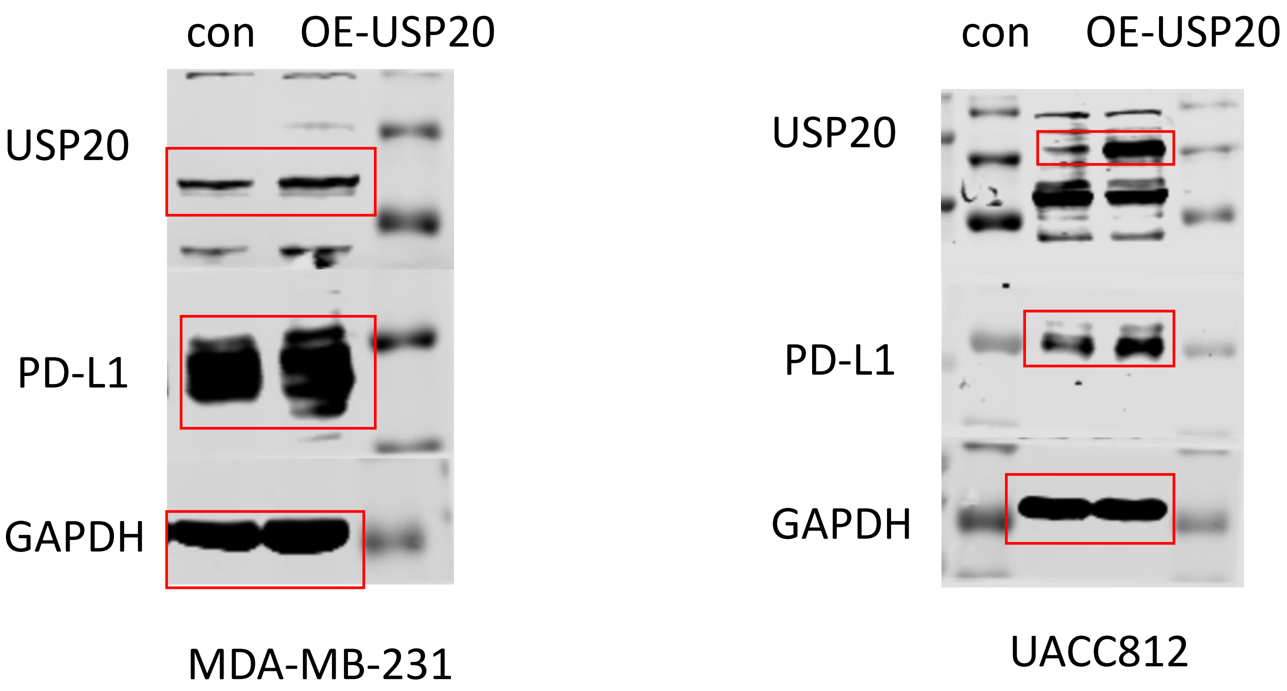
con
OE-USP20
USP20
PD-L1
GAPDH
MDA-MB-231
con
OE-USP20
USP20
PD-L1
GAPDH
UACC812

Figure 3I

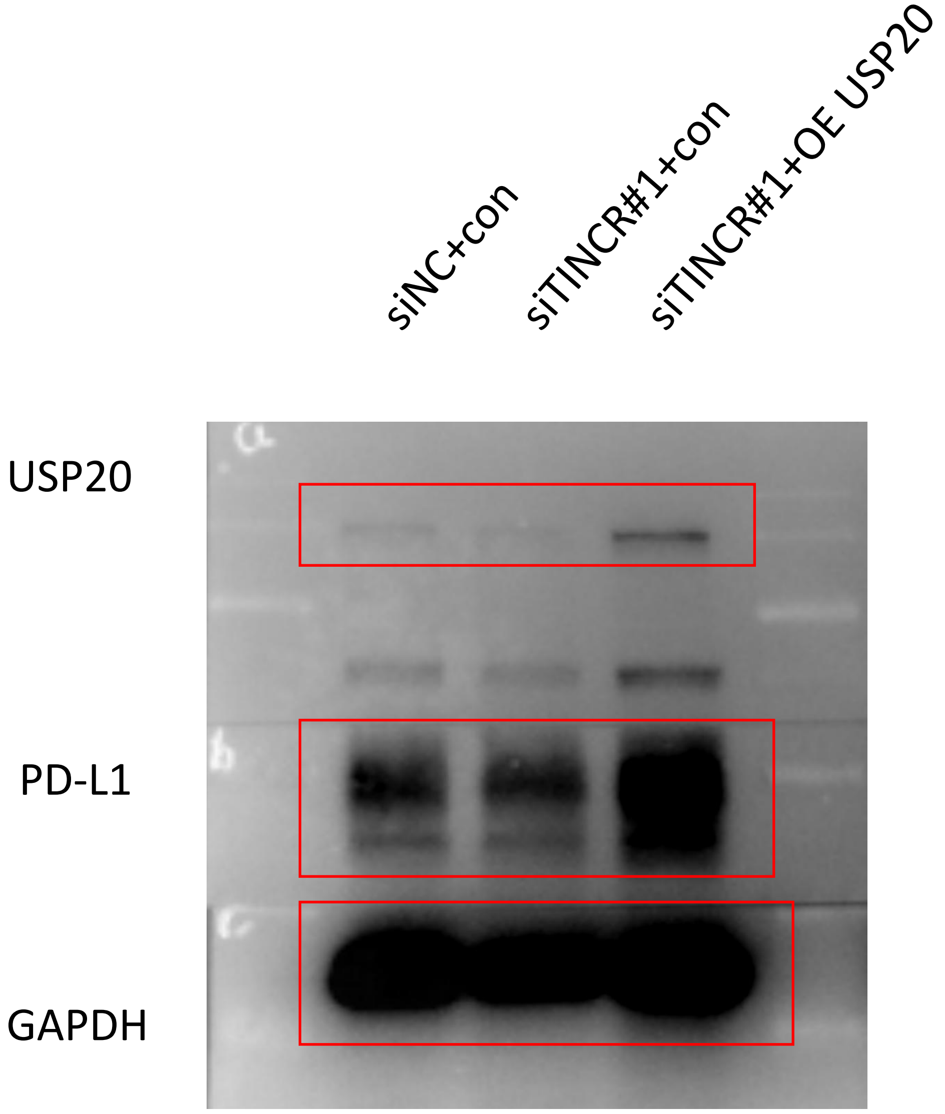

Figure 3J

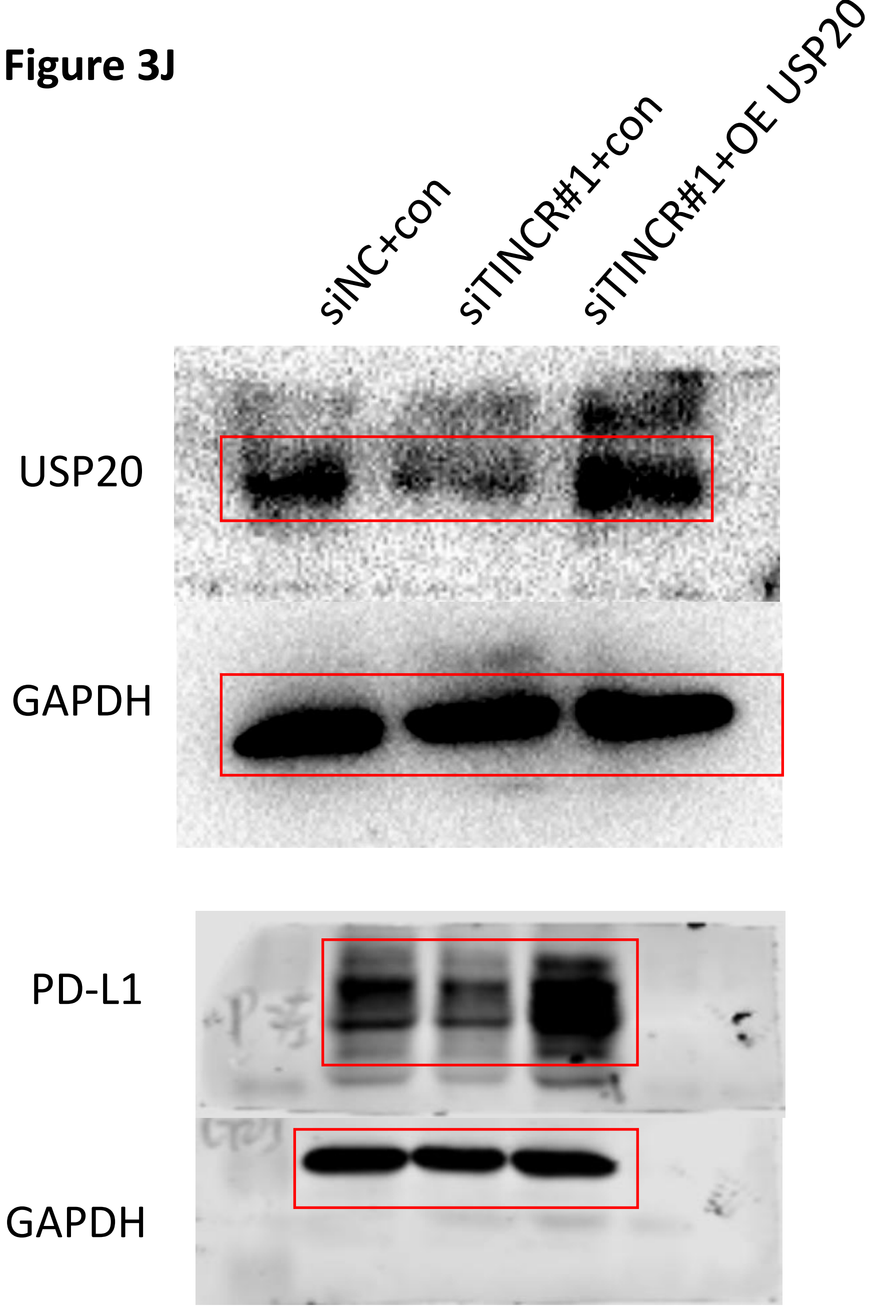

Figure 3K

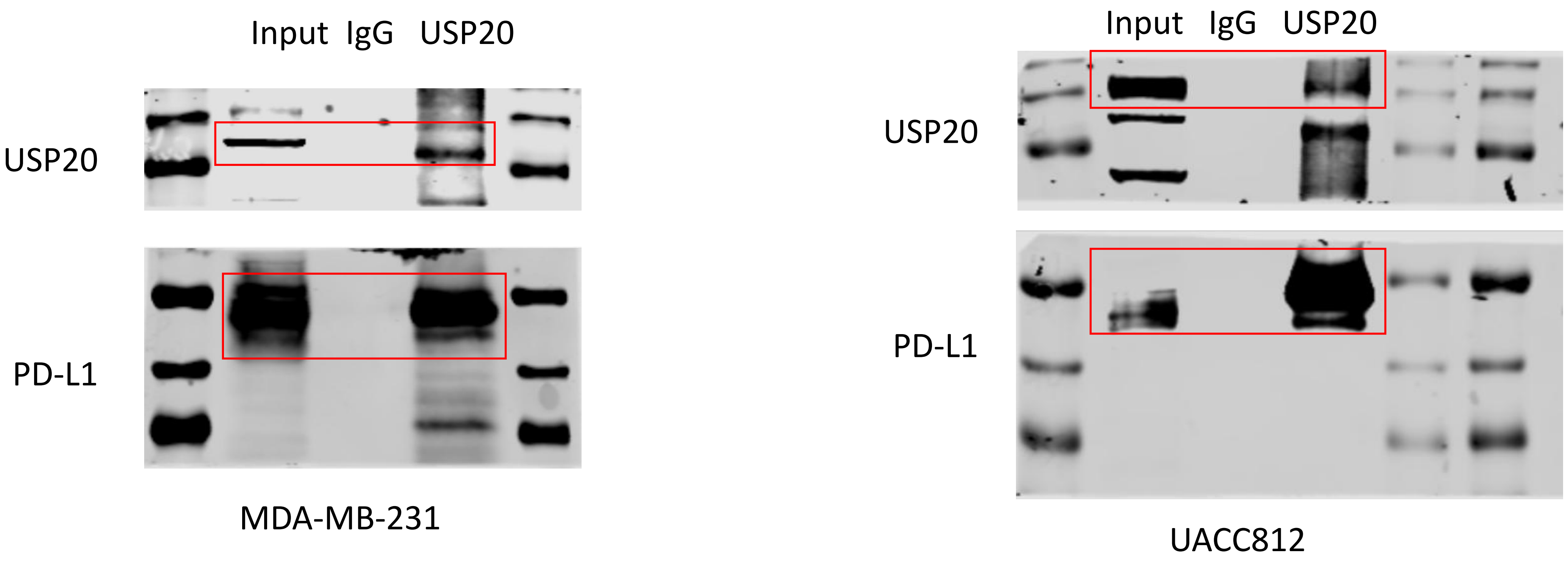
Input
IgG
USP20
USP20
PD-L1
MDA-MB-231
Input
IgG
USP20
USP20
PD-L1
UACC812

Figure 3L

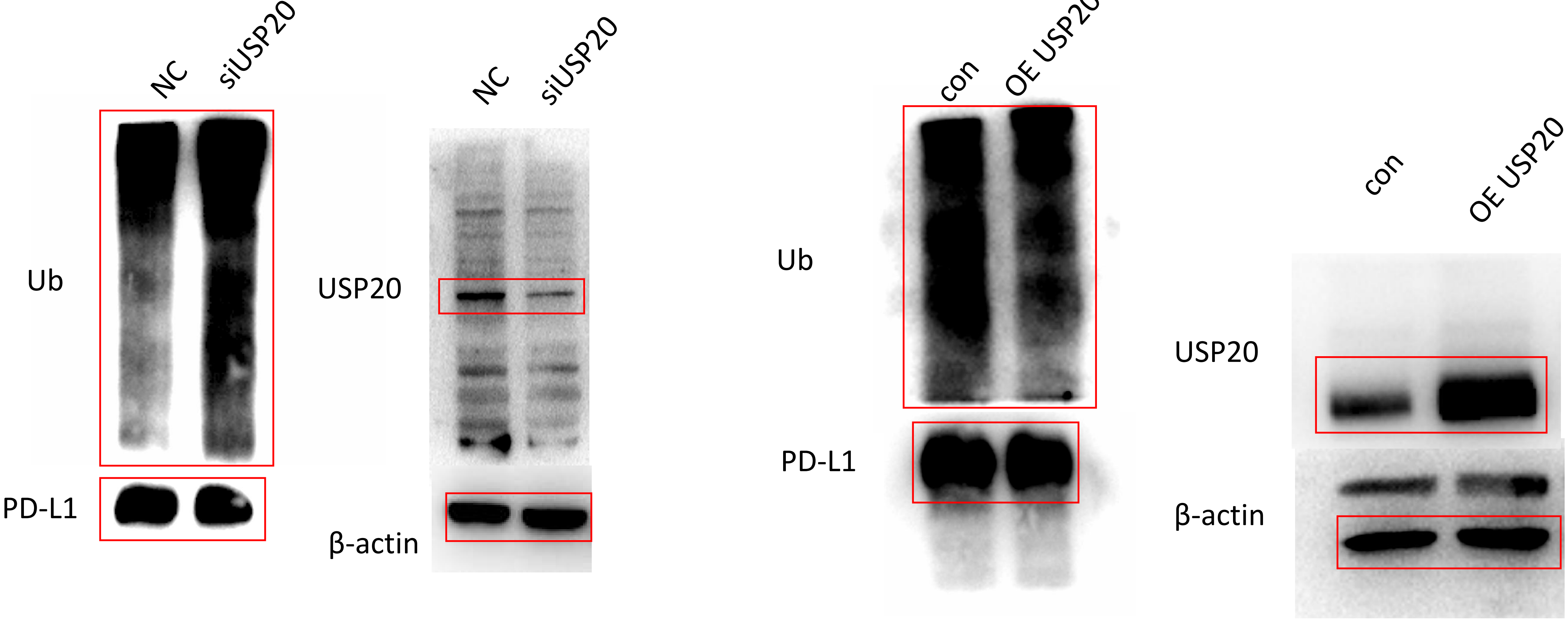
NC
siUSP20
Ub
PD-L1
NC
siUSP20
USP20
β-actin
con
OE USP20
Ub
PD-L1
con
OE USP20
USP20
β-actin

Figure 4F

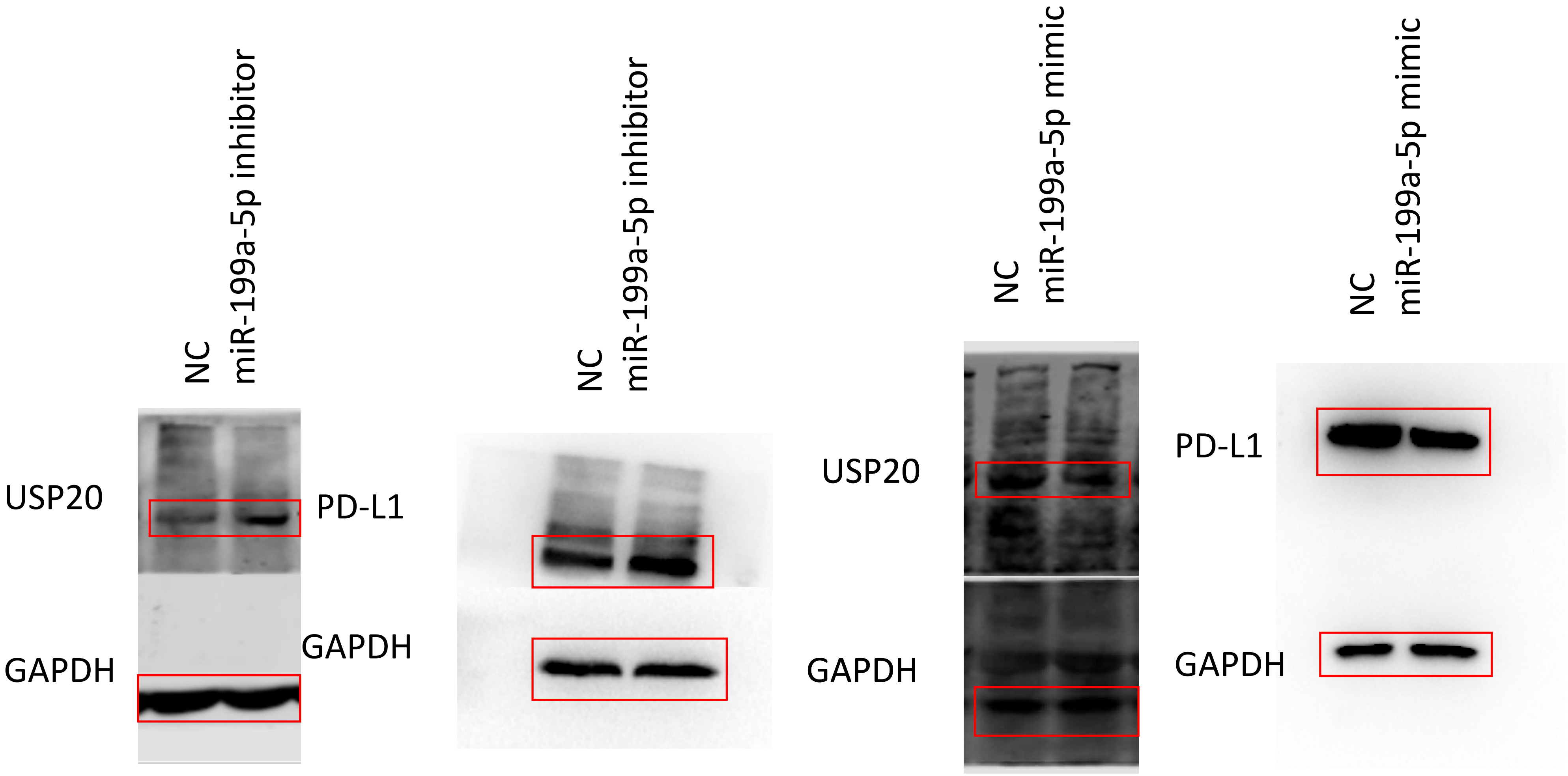

MDA-MB-231

Figure 4F

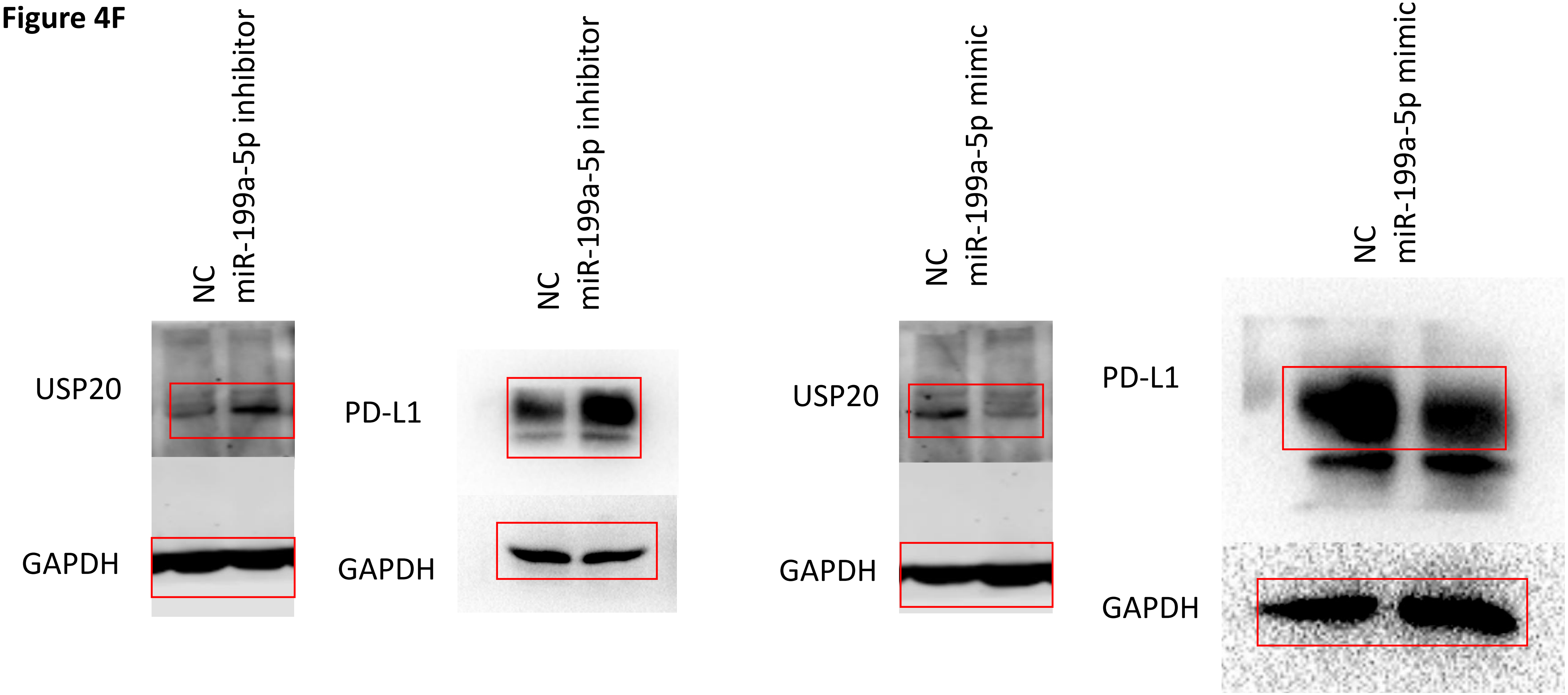

UACC812

Figure 4G

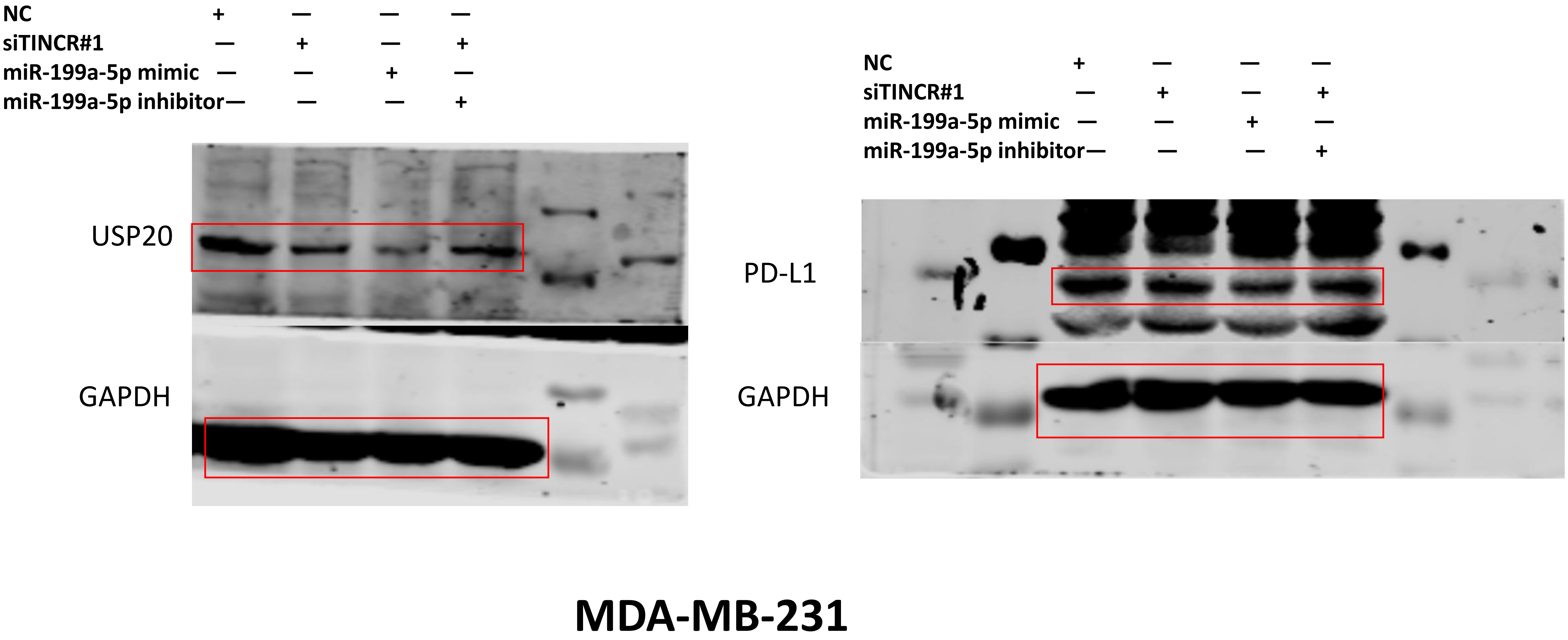
NC + — — —
siTINCR#1 — + — +
miR-199a-5p mimic — — + —
miR-199a-5p inhibitor — — — +
USP20
GAPDH
NC + — — —
siTINCR#1 — + — +
miR-199a-5p mimic — — + —
miR-199a-5p inhibitor — — — +
PD-L1
GAPDH
MDA-MB-231

Figure 6G

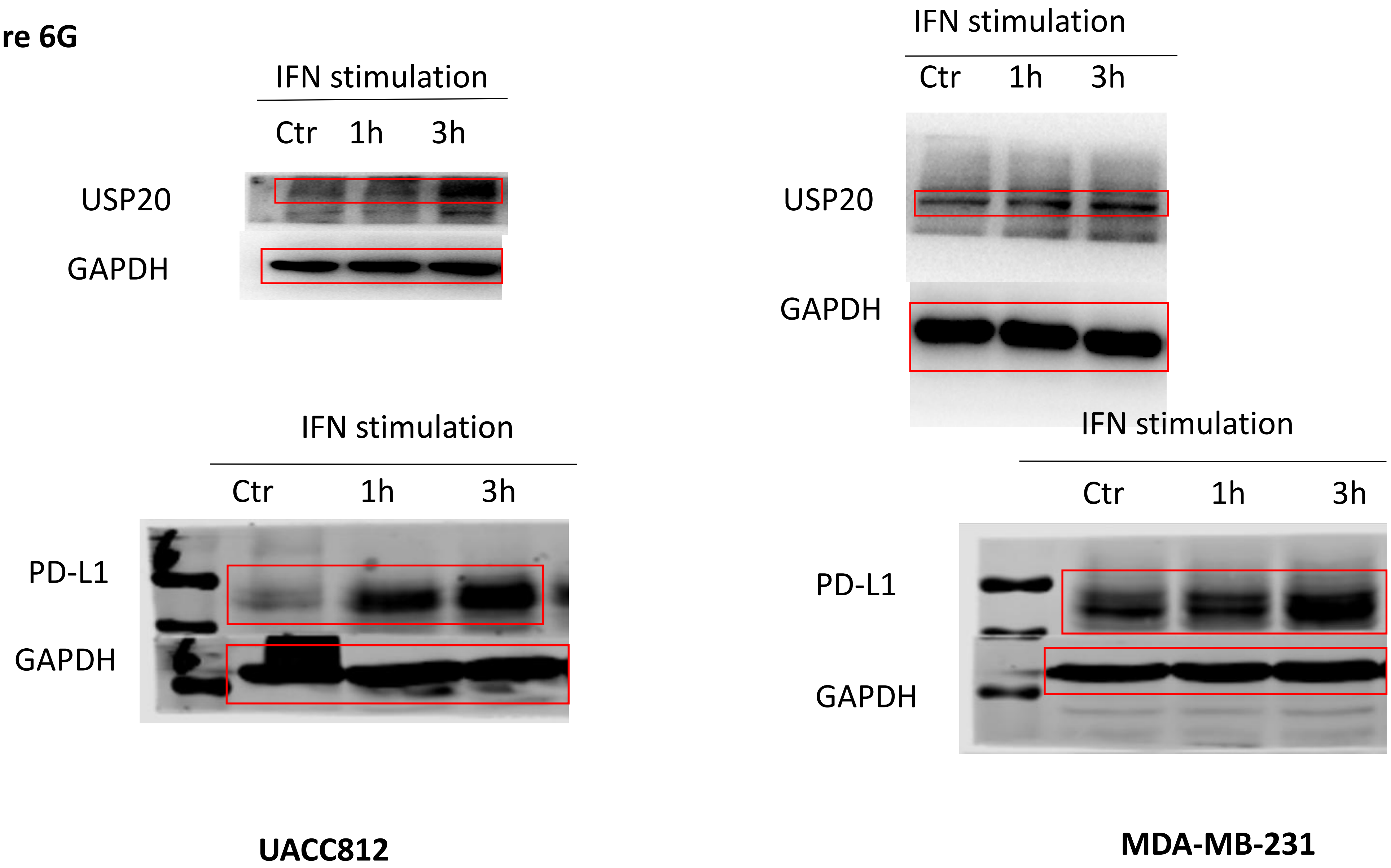
IFN stimulation
Ctr
1h
3h
USP20
GAPDH
IFN stimulation
Ctr
1h
3h
PD-L1
GAPDH
UACC812
IFN stimulation
Ctr
1h
3h
USP20
GAPDH
IFN stimulation
Ctr
1h
3h
PD-L1
GAPDH
MDA-MB-231

Figure 6I

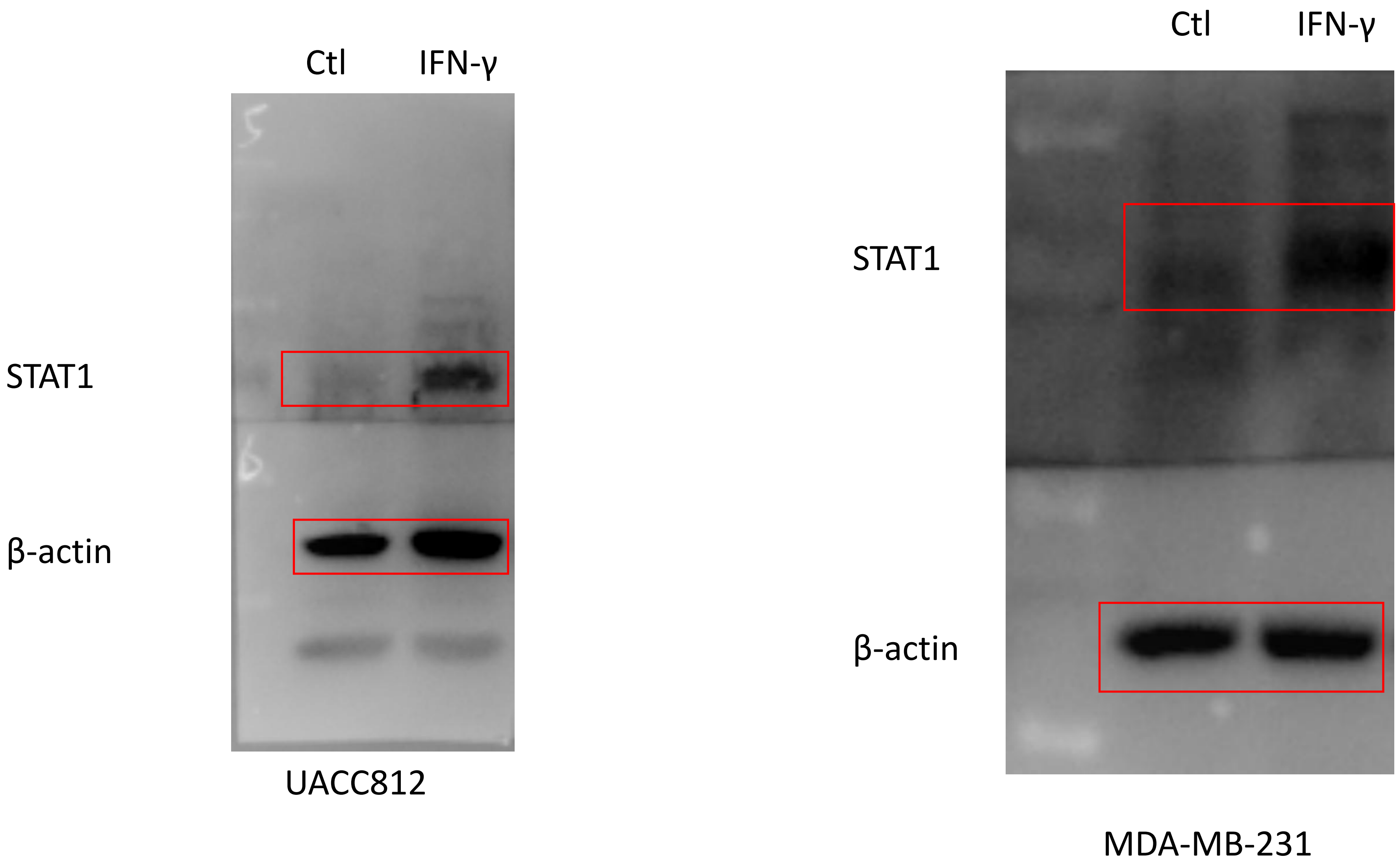
Ctl
IFN-γ
STAT1
β-actin
UACC812
Ctl
IFN-γ
STAT1
β-actin
MDA-MB-231

Figure 6J

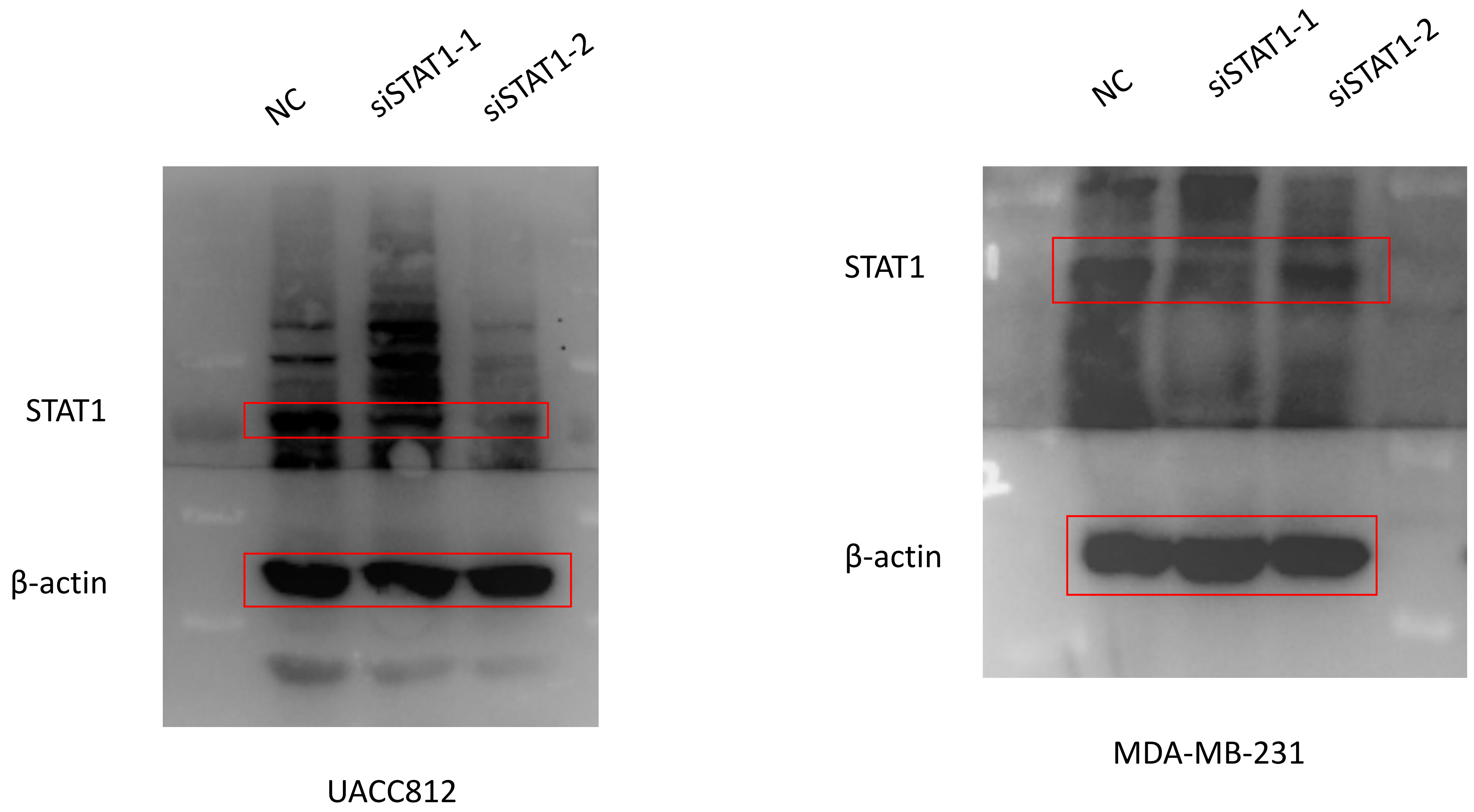

NC
siSTAT1-1
siSTAT1-2
STAT1
β-actin
UACC812
NC
siSTAT1-1
siSTAT1-2
STAT1
β-actin
MDA-MB-231

Figure 6M

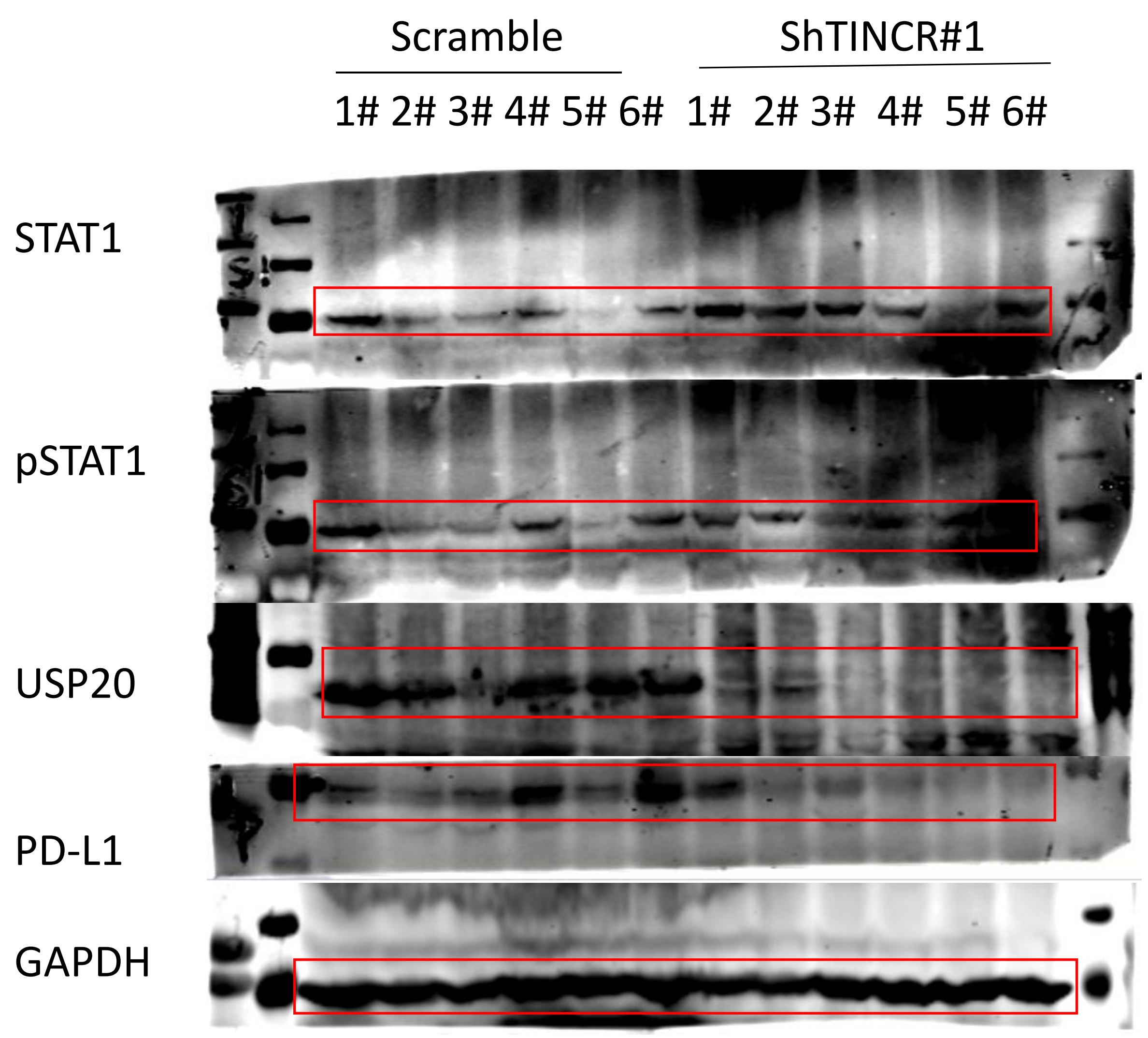

Scramble
ShTINCR#1
1# 2# 3# 4# 5# 6# 1# 2# 3# 4# 5# 6#
STAT1
pSTAT1
USP20
PD-L1
GAPDH

Supplementary Figure 2

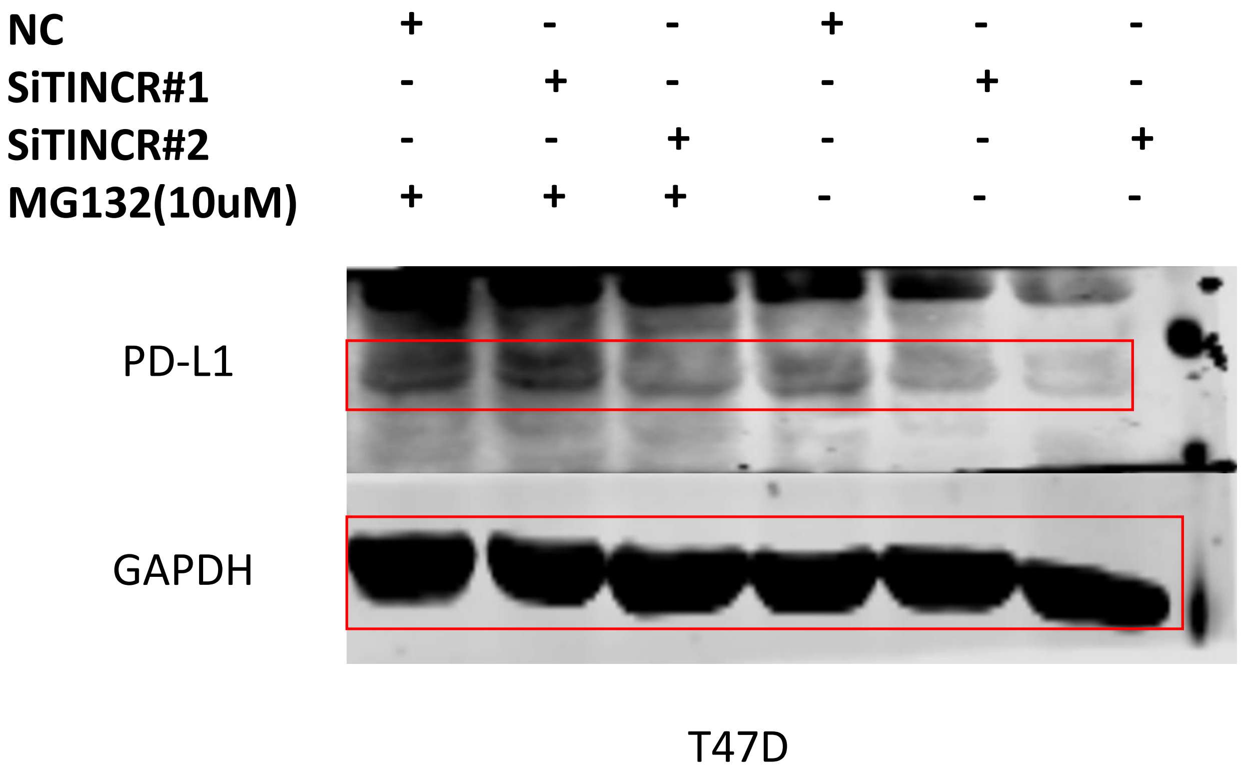

Supplement: Supplementary file 13 — Revised-western blot original data [file 41419_2023_5609_MOESM13_ESM.pdf]
